# Can *Fusobacterium* utilize nucleomodulins in the pathophysiology of CRC?

**DOI:** 10.3389/fcimb.2025.1644443

**Published:** 2025-10-30

**Authors:** Swadha Anand, Sania Korgaonkar, Chandrani Bose

**Affiliations:** TCS Research, Tata Consultancy Services Limited, Pune, Maharashtra, India

**Keywords:** *Fusobacterium nucleatum*, CRC, pathogenesis, host-pathogen interactions, nucleomodulins

## Abstract

*Fusobacterium nucleatum* (*Fn*) has been appositely termed as “oncobacterium” owing to its high abundance in colorectal tumor tissues throughout the course of tumorigenesis. However, beyond FadA and Fap2, there is a limited understanding of its virulence factors and mode of pathogenesis. Latest studies indicate an association of host genetic and epigenetic modifications with the degree of *Fn* colonization in tumor tissues. These changes are implicated in rendering the microenvironment more conducive for progression to later stages of tumorigenesis. Recent reports suggest the involvement of strains belonging to Clade C2 within *Fn* subspecies *animalis* (*Fna* C2) in colorectal cancer (CRC) that might have the ability to influence host homeostasis. In this study, we focus on the identification of candidate “nucleomodulins (NMs)”, which are nucleus-targeting effector proteins in *Fna* using various *in silico* sequence, structural and molecular dynamics simulation analyses. The results suggest the presence of a set of NMs in *Fna*, possessing a classical nuclear localization signal (cNLS), which can potentially bind to importin. This catalog of candidate NMs would help in designing experiments toward exploring their potential role in modulating host gene expression and epigenetics by entering the nucleus.

## Introduction

In recent years, *Fusobacterium nucleatum* (*Fn*), an anaerobic Gram-negative bacterium, has gained prominence for its role as “oncobacteria” in colorectal cancer (CRC) (Brennan and Garrett, 2019). An opportunistic oral pathogen, *Fn* translocates to the lower gastrointestinal tract and is heavily implicated in modulating gene expression in the tumor microenvironment (TME) (Brennan and Garrett, 2019; Kim et al., 2023). An increase in the abundance of *Fn* is observed throughout the course of tumorigenesis, from one of the earliest precursors of CRC, aberrant crypt foci (ACF) to metastasized tumors (Kostic et al., 2013; Flanagan et al., 2014; Bullman et al., 2017). Recent genomic analyses have revealed an enrichment of strains belonging to Clade C2 of *Fn* subspecies *animalis* (*Fna* C2) in CRC tissues, a subspecies not prevalent in the oral niche (Zepeda-Rivera et al., 2024). Prior to this study, most of the experiments used other subspecies of *Fn* such as *Fn* subspecies *nucleatum* (*Fnn*) ATCC 25586 and *Fnn* ATCC 23726 as model organisms (Zepeda-Rivera et al., 2024), and their implication in CRC development as well as progression was observed in multiple studies (Abed et al., 2016; Yu et al., 2017; Zepeda-Rivera et al., 2024). In addition to CRC, different subspecies of *Fn* have also been associated with changes in host gene expression and signaling pathways involved in the manifestation of oral disease conditions like periodontitis, plaque formation, and oral cancer (Chen et al., 2022; Ponath et al., 2022; Galaski et al., 2024; Wolf et al., 2025). Sequencing of these subspecies has revealed significant genetic dissimilarities between the five subspecies for each of them to be considered as a distinct species (Kook et al., 2017; Zepeda-Rivera et al., 2024). To date, widely discussed virulence factors of *Fn* include adhesins FadA and Fap2 that facilitate bacterial adhesion to the host cells by activating E-cadherin/β-catenin pathways and recognizing overexpressed Gal-GalNAc, respectively (Abed et al., 2016; Meng et al., 2021). Furthermore, there have been numerous studies highlighting the association of genetic and epigenetic modifications in different cancers with the intratumoral load of *Fn (* Kostic et al., 2013; Xia et al., 2020; Udayasuryan et al., 2024). Nonetheless, there is still a considerable gap in understanding the potential entities from *Fn*, which might be associated with manipulating the host homeostasis.

The role of bacterial effector proteins in host microbiome interactions and the pathophysiology of disease conditions has been elucidated in many pathogens (Lee et al., 2012; Moon et al., 2012a; Hanford et al., 2021). One category of these bacterial effector molecules includes nucleomodulins (NMs) that target the host nucleus (Hanford et al., 2021). Secretion of NMs by bacterial pathogens is one of the many tactics of bacteria to outwit the host immune responses (Bierne and Cossart, 2012; Hanford et al., 2021). As the name suggests, NMs have the potential to translocate to the host nucleus and modify a multitude of cellular processes such as cell cycle arrest, epigenetic modifications, and regulation of gene expression in an attempt to survive and replicate within the host environment (Hanford et al., 2021). These NMs utilize various mechanisms, usually mimicking eukaryotic nuclear targeted proteins, to enter the guarded nucleus by surpassing nuclear envelope (Bierne and Cossart, 2012; Hanford et al., 2021). These mechanisms primarily include harboring a nuclear localization signal (NLS) on its sequence or hijacking eukaryotic proteins that are directed towards the host nucleus (Bierne and Cossart, 2012). NLSs are classified broadly into two types, classical NLS (cNLS) and non-classical NLS (ncNLS) (Görlich and Kutay, 1999; Kosugi et al., 2009). Depending on the type of signal, the import of NMs inside the nucleus is facilitated by importins, which belong to the class of proteins known as “karyopherins” (Cooper, 2000). cNLS consists of amino acid residues such as arginine (R) and lysine (K) and are further categorized as “monopartite” (MP) and “bipartite” (BP) signals. MP signals consist of four to eight basic amino acids while BP signals consist of two clusters of basic amino acids separated by a linker amino acid sequence (Kosugi et al., 2009). Proteins with unusual NLS that are not similar to canonical cNLS are referred to as ncNLS (Görlich and Kutay, 1999; Cingolani et al., 2002; Kosugi et al., 2009).

**Figure 1:**
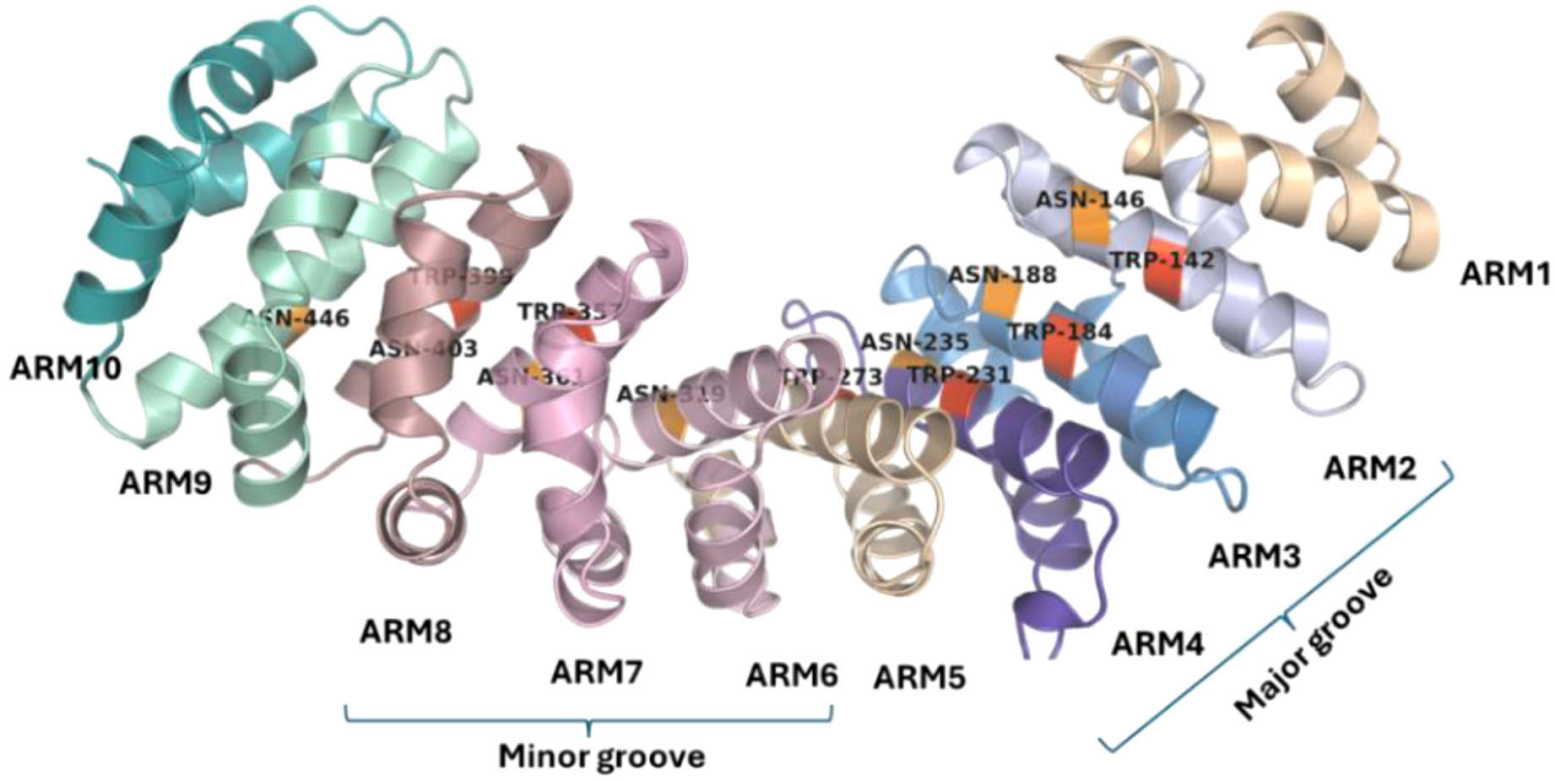
Structure of importin α. The 10 Armadillo repeats in importin α show the presence of conserved tryptophan residues (red) and asparagine residues (orange). The ARM repeats 2–4 form the major groove of the importin α while the ARM repeats 6–8 form the minor groove.

In classical nuclear import, importin α recognizes the NLS followed by its attachment to the cargo and presents it to importin β, which transports this ternary complex into the host nucleus (Görlich and Kutay, 1999). The type of residue interactions within the importin–cNLS complex is conserved as indicated by structures determined for yeast, mouse, and human importins (Kosugi et al., 2009). The C-terminal region of the importin α consists of 10 Armadillo (ARM) repeats each composed of three α helices (Sankhala et al., 2016) (**Figure 1**). ARM repeats have conserved Trp and Asn residues involved in forming interactions with cNLS (Kosugi et al., 2009; Sankhala et al., 2016). The Trp residues form a stacking array, which can form stacking interactions with the carbon atoms in Arg and Lys residues in cNLS while the Asn residues form interactions with polar residues as well as the backbone atoms in cNLS (Kosugi et al., 2009; Oka and Yoneda, 2018). Further basic residues in cNLS form interactions like salt bridges, hydrogen bonds, and electrostatic interactions with negative residues lining importin binding sites (Sankhala et al., 2016). The Asp192 of importins has been shown to interact with a Lys residue in cNLS in most solved structures of importins bound to cNLSs (Sankhala et al., 2016).

In this *in silico* study, we have explored the potential effectors in *Fna* that can work as NMs through various sequence and structural analysis tools and techniques. Interactions of candidate NMs, identified in this study, with the major or minor groove of importin α protein are delineated using protein–protein docking and molecular dynamics (MD) simulations. The study proposes a paradigm in which proteins belonging to different functional categories like nucleases, transcription factors, and DNA/RNA binding proteins from *Fn* might possess an ability to enter the host nucleus and might contribute in the pathophysiology of CRC.

## Methods

### Selection of *Fusobacterium nucleatum* strain for analysis

Genomic sequences of the strains belonging to *Fna* were analyzed to identify a representative strain on which further analysis can be performed. Nucleotide sequences of 65 genomes of *Fna* deposited under BioProject PRJNA549513 (Zepeda-Rivera et al., 2024) were downloaded from the National Center for Biotechnology Information (NCBI) Nucleotide database (https://www.ncbi.nlm.nih.gov/nucleotide/) in nucleotide FASTA format. In order to select a representative *Fna* strain (implicated in CRC) for further analysis, *Fna* Clade 2 (*Fna* C2) was considered owing to its enrichment in CRC tumors (Zepeda-Rivera et al., 2024). The nucleotide sequences of the strains belonging to *Fna* C2 were analyzed using the FastANI tool (https://github.com/ParBLiSS/FastANI) in order to evaluate pairwise average nucleotide identity (ANI) and subsequent selection of the representative strain.

### Identification of nucleomodulins through prediction of cNLS in representative strain

Amino acid sequences corresponding to proteome of the selected strain were downloaded from the NCBI Genome database in FASTA format. Publicly available tools with distinct algorithms were used for the identification of potential cNLS on proteins. These include NLStradamus (Nguyen Ba et al., 2009) (http://www.moseslab.csb.utoronto.ca/NLStradamus/), which operates on a simple HMM (Hidden Markov Model) algorithm; NucImport (Mehdi et al., 2011) (UQ eSpace), which is a combination of three Bayesian network models; and NucPred (Brameier et al., 2007) (https://nucpred.bioinfo.se/nucpred/), which utilizes regular expressions from NLSs and assigns a likelihood score using an ensemble model consisting of over 100 sequence-based predictors. All these tools provided scores in the range of 0 to 1, and the values were noted for each protein in the proteome of *Fna*. In order to arrive at a cutoff score that might be a good indicator of an actual cNLS, we utilized these tools for assessment of NLS sequences identified in experimentally characterized bacterial NMs (**Supplementary Table 1**). The results indicated that at least one of the three tools yielded a score of ≥0.6 for most of the experimentally characterized bacterial NMs with a cNLS (**Supplementary Table 1**). Thus, we have considered a cutoff score of 0.6, in at least one tool, to predict a probable set of NMs. A subset of the predicted NMs with a more stringent prediction score of ≥0.7 was further considered for detailed analysis. This subset of proteins was analyzed using NCBI BLASTp (https://blast.ncbi.nlm.nih.gov/Blast.cgi) for assessing their presence within the *Fn* genomes and across other bacteria, and analyzing their NLS sequence conservation.

### Analyzing binding of NM to importin α through in silico docking

The structure PDB ID 5HUY was obtained from RCSB Protein Data Bank (https://www.rcsb.org/) (RCSB PDB: homepage), which corresponds to an importin α bound to the NLS of a small terminase from HCMV. The modeled structures for the identified candidate NM proteins in *Fusobacteria* were obtained from the AlphaFold Protein Structure Database (https://alphafold.com/) (Varadi et al., 2024; Varadi et al., 2022; Jumper et al., 2021). Structures that were unavailable in the database were modeled using the AlphaFold monomer model with the DeepMind Colab Jupyter notebook with default settings (Jumper et al., 2021). The binding interaction between candidate proteins (in *Fna* possessing NLS) and importin α was analyzed using the HADDOCK 2.4 (High Ambiguity Driven protein–protein DOCKing) (Honorato et al., 2024) (https://rascar.science.uu.nl/haddock2.4/) web server with default parameters. HADDOCK deploys *ab initio* docking methods by incorporating data from known or predicted protein interfaces into ambiguous interaction restraints (AIRs) to guide the docking process. Both proteins were submitted in PDB format in the HADDOCK interface specifying the constraints for docking including active residues of importin α based on experimental sources and the NLS residues of candidate proteins identified in previous steps. The pocket residues from the major groove of importin α provided as constraints were Ser105, Trp142, Asn146, Ser149, Gly150, Thr155, Q181, Trp184, Asn188, Gly191, Asp192, Trp231, Asn228, Asn235, Arg238, Asn239, and Trp273. Furthermore, the modeled complexes were shortlisted based on the HADDOCK score, which accounts for the weighted sum of various energy components that differ with respect to each docking stage: rigid body (it0), semi-flexible refinement (it1), and explicit solvent refinement (water). The default values considered are as follows:

▪ HADDOCKscore-it0 = 0.01 Evdw + 1.0 Eelec + 1.0 Edesol + 0.01 Eair − 0.01 BSA
▪ HADDOCKscore-it1 = 1.0 Evdw + 1.0 Eelec + 1.0 Edesol + 0.1 Eair − 0.01 BSA
▪ HADDOCKscore-water = 1.0 Evdw + 0.2 Eelec + 1.0 Edesol + 0.1 Eair

where Evdw = van der Waals intermolecular energy, Eelec = electrostatic intermolecular energy, Edesol = desolvation energy, Eair = distance restraints energy [only unambiguous and AIR (ambig) restraints], and BSA = buried surface area (https://rascar.science.uu.nl/haddock2.4/). Following the visualization of the top four scoring complexes in https://www.pymol.org/, the most optimal complex was chosen for detailed analysis. These structures were further evaluated by calculating contact distances (default cutoff, 5 Å) using MAPIYA (Badaczewska-Dawid et al., 2022) (https://mapiya.lcbio.pl/). Furthermore, to investigate the probable impact of mutation on the interaction between importin α and RBPL34, the conserved Asp192 was substituted by Ala. The mutated structure was then modeled using Swiss-Model (https://swissmodel.expasy.org/) (Waterhouse, 2018) with the structure corresponding to *Mus musculus* importin alpha2 (PDB ID: 3TPO) as a template.

### MD simulations of protein–protein complexes

#### System preparation

The protein complex structure (unmutated and mutated) obtained from HADDOCK was used and the missing hydrogen atoms were added, and protonation states of titratable residues were assigned at physiological pH (7.0) using the PROPKA module integrated within the preparation workflow. The topology and parameters of the protein complex were generated using the AMBER ff14SB force field. The system was solvated explicitly in a cubic box of TIP3P water molecules, extending at least 10 Å from any protein atom. Sodium and chloride counter-ions were added to neutralize the system and achieve an ionic strength of 0.15 M.

#### Energy minimization and equilibration

Initial steric clashes and unfavorable contacts were removed by using 10,000 steps of Steepest descent minimization. The system was then equilibrated in two stages. First, the system was heated gradually from 0 to 300 K over 500 ps under constant volume (NVT) conditions using a Berendsen thermostat with a collision frequency of 2 ps^-^¹. In the second stage, equilibration was carried out for 1 ns under constant pressure (NPT) conditions using a Berendsen barostat at 1 atm with a relaxation time of 2 ps.

#### Production molecular dynamics simulation

The production MD simulation was performed for 40 ns with an integration time step of 1 fs (0.001 ps) using the Leapfrog integrator. The particle mesh Ewald (PME) method was applied to treat long-range electrostatics with a real-space cutoff of 10 Å. Van der Waals interactions were truncated at 10 Å with a switching function applied from 8 Å. All covalent bonds involving hydrogen atoms were constrained using the LINCS algorithm. Periodic boundary conditions were applied in all directions.

Temperature was maintained at 300 K with a Langevin thermostat, and pressure was maintained at 1 atm with an isotropic Parrinello–Rahman barostat. Trajectory snapshots were saved every 10 ps for subsequent analysis. Two replicates of the simulation were performed with different random seeds.

#### Simulation environment and analysis

All simulations were performed using the GROMACS, 2022.3 package (Spoel et al., 2005). The changes in the importin during the simulations were assessed by calculating the radius of gyration using the gmx gyrate command in GROMACS. Further distances between the important residues in importin and NLS binding that form the electrostatic and hydrophobic linkages were also calculated using the gmx distance command in GROMACS. Secondary structures of the predicted NLS region were also analyzed in GROMACS.

## Results

### Selection of representative strain of *Fusobacterium nucleatum*

In order to identify potential nucleomodulins in *Fna*, a representative genome was selected based on the ANI metric. ANI values below 90% indicate that the genomes are from different species, while values greater than 95% suggest that the genomes belong to the same species (Rodriguez-R et al., 2023). As mentioned earlier, the reclassification of *Fn* subspecies has been proposed, as the ANI values when compared between subspecies are below 95%, indicating significant genomic heterogeneity (Kook et al., 2017). This is evident in the case of *Fna* genomes including Clade 1 (C1) and Clade 2 (C2), which generated ANI values ranging from 91% to 99% when compared pairwise using FastANI. Based on these values, *Fna* strain SB031 (2,432,266 bp, GenBank Assembly ID: GCA_037897425.1), isolated from CRC tissue, which exhibited ANI > 96% with other members of *Fna* C2, was selected for subsequent analysis (**Supplementary Figure 1**).

### Identification of nucleomodulins from *Fna* SB031

As discussed in the Methods section, multiple tools are available to identify cNLS within a protein (NLStradamus; Brameier et al., 2007; Mehdi et al., 2011). NLStradamus, Nucimport, and NucPred were utilized for the prediction of cNLS to identify potential NMs within the proteome (2,204 proteins) of *Fna* SB031. The results showed the presence of potential cNLS in 330 proteins with a score ≥0.6 in one or more tools (scores from 3 tools and predicted NLS sequence details are provided in **Supplementary Table 2**).

We selected the five proteins that showed a score greater than 0.7 for all the three tools for a detailed analysis of their probable interactions with the importin protein (**Table 1**). Additionally, we also analyzed the secondary structure of the experimentally characterized NMs and their corresponding cNLSs using JPred4 (https://www.compbio.dundee.ac.uk/jpred/) (Drozdetskiy et al., 2015) in order to understand the potential structural features that bacterial cNLSs can adopt, and the results are provided in **Supplementary Figure 2**. A detailed sequence and structural analysis on NLS of the predicted NMs is provided below.

**Table 1:** Potential nucleomodulins in *Fna* SB031.

GenBank accession IDs	Predicted NMs	NI score	NP score	NS score	NI-predicted NLS	NP-predicted NLS	NS-predicted NLS
WYD17043	50S ribosomal protein L34 (RBPL34)	0.99	0.82	0.99	NGRKVLKRRRVRGRAKLSA	QRKRKKDH	QRKRKKDHGFRARMSTKNGRKVLKRRRVRGRAKLS
WYD17513	Transposase (Tnp)	0.91	0.88	0.97	EKTGKFKKKKRFGKSLSNRA	QRRSKKTEMSEKTGKFKKKKRFG	QRRSKKTEMSEKTGKFKKKKRFGKSL
WYD16145	Type II CRISPR RNA-guided endonuclease Cas9	0.97	0.75	0.95	NSRRRLKRRKWRLNLLEEIF	NSRRRLKRRKWRL	KTAAERRVQRNSRRRLKRRKWR
WYD16130	YgiQ family radical SAM protein	0.84	0.74	0.74	ANYTATKKRRQQDDFTPGGV	TATKKRRQQDDFTPGG	KKQKQKDIVTEKRRK
WYD16313	Replication initiator protein A	0.85	0.81	0.74	ENLKLIKKKKRYKNSSVYYL	LIKKKKRYKNSSVYYLENVG	KKNTISNALKELENLKLIKKKKRY

NI, NucImport; NP, NucPred; NS, NLStradamus.

Predicted NMs ≥0.7 for tools NucImport, NucPred, and NLStradamus. The cNLS sequences predicted by the tools have also been indicated.

### Ribosomal protein L34 as a potential nucleomodulin in *Fusobacteria*

The results of NLS prediction tools indicated the set of residues 26–44 as a potential cNLS NGRKVLKRRRVRGRAKLSA in ribosomal protein L34 (RBPL34) from *Fusobacterium*. The sequence is conserved across the genomes of all strains of *F. nucleatum* subsp. *animalis*, *F. nucleatum* subsp. *polymorphum (Fnp)*, and *F. nucleatum* subsp. *vincentii (Fnv)* often implicated in CRC and oral diseases. Furthermore, a sequence-based search for homologs within complete bacterial genomes indicated that the same NLS is not observed in *F. varium, F. necrophorum*, and *F. ulcerans*. Interestingly, on further analysis, it was found that the C-terminal region of RBPL34 from these species also shows a high score for the presence of NLS within their sequences. Therefore, these proteins may also show nuclear localization probability but utilizing a different cNLS sequence. Analysis of RBPL34 homologs in bacteria other than *Fusobacterium* indicated that despite a high sequence similarity in the N-terminal region, the C-terminal part (comprising the identified NLS sequence in *Fusobacterium*) of the sequences shows divergence and, in some cases, the presence of a different NLS sequence from the one observed in *Fna*. Earlier studies show a higher abundance of *Fusobacterium* subspecies like *Fnn*, *Fnv*, and *Fnp* in these oral disease conditions and indicate their role in influencing the host disease microenvironment and association with changes in gene expression and signaling pathways (Chen et al., 2022; Galaski et al., 2024; Wolf et al., 2025). The presence of cNLS in RBPL34 from other subspecies of *Fusobacterium* indicates that they might play a role in influencing gene expression in oral conditions like plaque formation, periodontitis, and oral cancer. Additionally, experimental studies have also implicated certain strains of subspecies *Fnn* in the development and manifestation of CRC (Abed et al., 2016; Yu et al., 2017). Thus, the presence of candidate NLS sequences in RBPL34 of these species might indicate their potential role in explaining the association of *Fn* with different disease mechanisms.

As mentioned above, the cNLS-containing proteins need to bind to the importin α major groove to enter the nucleus (Sankhala et al., 2016). In order to further corroborate if the identified cNLS in the protein shows a propensity to interact with importin α protein, we docked the model built using AlphaFold for RBPL34 from *Fna* SB031 with importin α protein (PDB ID: 5HUY) using HADDOCK 2.4 (as described in the Methods section). Most of the experimentally characterized structures available for importin only show the binding of the NLS from the candidate nuclear protein without accounting for the remaining part of the protein. Recent studies have indicated that the importin–NLS binding depends on the context within the protein in addition to the NLS itself (Friedrich et al., 2006). Thus, we chose to utilize the whole structure instead of only docking the cNLS to the importin structure. We analyzed the energy landscape and postures for RBPL34 obtained in the docking clusters and selected the best cluster for further analysis (**Supplementary Figure 3**, **Supplementary Table 3**) with a HADDOCK score of −125.53 (a score with a more negative value is considered better).

**Figure 2:**
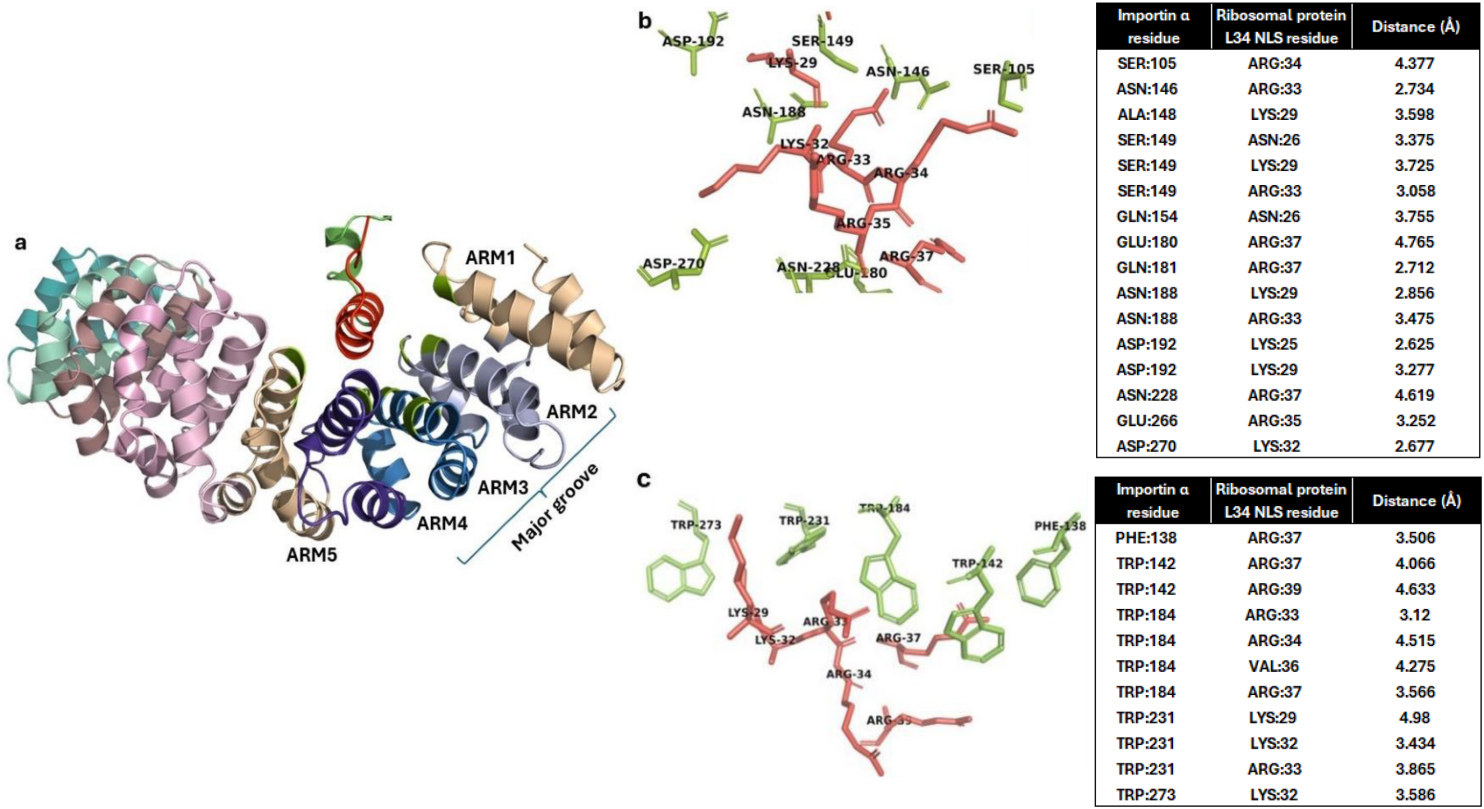
Interaction of importin α with *Fna* SB031 ribosomal protein L34 (RBPL34). **(a)** Interaction of NLS in RBPL34 from *Fusobacterium* with the ARM repeats 2, 3, and 4 of importin α. **(b)** The electrostatic, salt bridge, and hydrogen bond interactions within the docked complex of RBPL34 and importin α are depicted. The interacting residues from importin are depicted in green while those from RBPL34 are depicted in red. **(c)** The cation–pi and hydrophobic interactions within the docked complex of RBPL34 and importin α are depicted. The interacting conserved tryptophan residues from importin are depicted in green while the corresponding interacting residues from RBPL34 are depicted in red.

We further analyzed the obtained docked complex to check if the contacting residues between importin and RBPL34 are similar to the interactions with Trp and Asn residues as well as with Asp192 in importin as have been reported in experimentally characterized importin NLS structures (Sankhala et al., 2016). The helix of the RBPL34 protein containing the predicted NLS residues 26–44 is seen to interact with ARM repeats 2, 3, and 4 in the major groove of importin α (**Figure 2a**). **Figure 2b** shows the electrostatic interactions between the NLS and importin in the docked complex. As observed in the experimentally resolved structures, the conserved Asn146 and Asn188 of ARM 2 and 3 show an electrostatic/hydrogen bonding interaction with polar residues Lys29 and Arg33 in the cNLS of RBPL34. Furthermore, the conserved Asp192 in importin shows salt bridge formation with Lys25 and Lys29 on the NLS of RBPL34. **Figure 2c** indicates that the hydrophobic Trp array from ARM 2, 3, and 4 (corresponding to Trp142, Trp184, and Trp231) shows cation–pi interactions with the long carbon side chains of basic residues in the cNLS (including Lys29, Lys32, Arg33, Arg34, and Arg39) of RBPL34.

### Assessing conformational dynamics of the importin–RBPL34 complex using MD simulations

In order to assess the viability of the identified interactions as well as possible conformational dynamics of the importin complex with RBPL34, we performed explicit solvent MD simulations (see the Methods section). The results corresponding to radius of gyration (for both simulation replicates) showed the reduction in the first 2 ns followed by the stabilization of the Rg value (**Supplementary Figure 4A**). To further understand the changes in the secondary structure of the NLS sequence, we analyzed the trajectory using DSSP, and the results indicated that the secondary structure fluctuated between helix and loops for most residues although we observe higher occurrence of loop structures towards the end of the simulation (**Supplementary Table 4**). These results indicated that the secondary structure of different NLS residues switches between loops and helices, indicating conformational changes in the NLS region as well (**Supplementary Figure 4B**).

**Figure 3:**
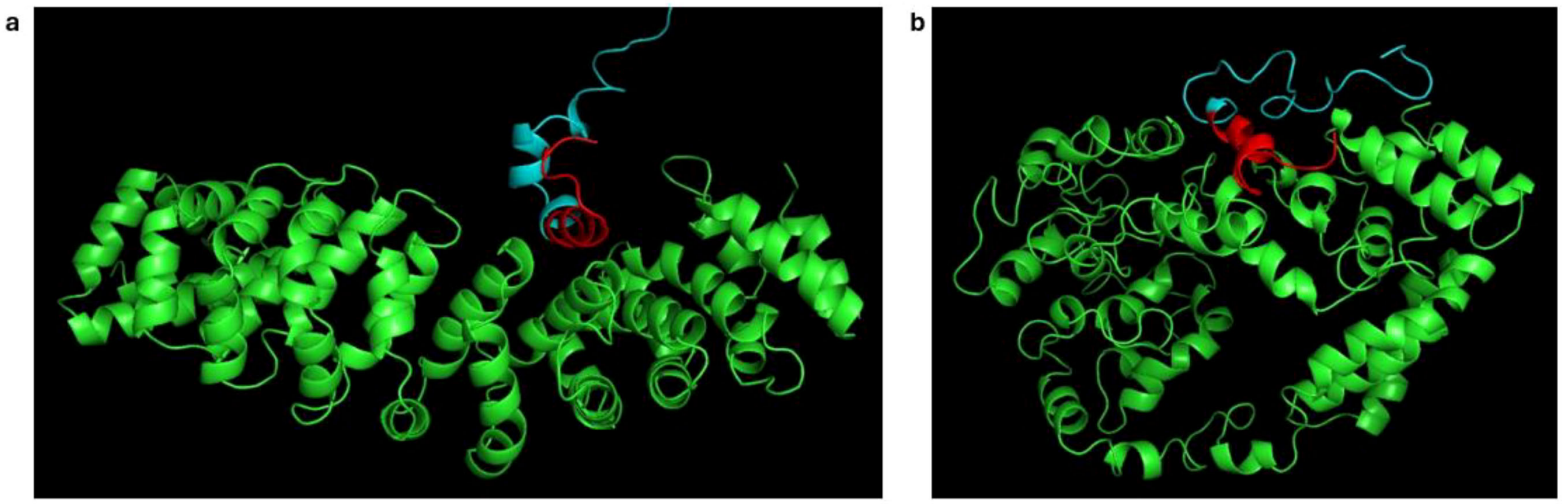
Changes in conformation of importin ARM helices during molecular dynamics simulation. **(a)** Snapshot at 50 ps showing the conformation of ARM helices (green). **(b)** Snapshot at 2 ns, highlighting rearrangement. The predicted NLS is shown in red.

On visualization, it was observed that the ARM helices rearranged, which might be conformational changes to accommodate the whole RBPL34 protein (**Figure 3**). Earlier studies have shown that the Armadillo helices of importin show a spring like movement to accommodate cNLS and the protein cargo (Sankhala et al., 2017). Studies have further shown that the conformational changes are more prominent in case of complete cargo binding since the ARM helices 2–4 show a movement to accommodate the larger protein without steric clashes and with favorable interactions. Thus, the changes in radius of gyration as well as the overall orientation of helices observed on visualization might be an indication of these interactions.

We observed that despite the changes in importin helices, the interactions between the conserved acidic patch containing Asp192 and the basic residues remained intact. Thus, we further analyzed the change in distance between these residues to assess if the interaction between the importin and NLS remained stable across both replicates of the MD simulation. Previous studies indicate that the residue Asp192 is conserved in importin α and located in the acidic patch of ARM helix responsible for stabilizing the basic residues within the NLS. During the simulation studies, it was observed that the Asp192 formed persistent electrostatic contacts with both Lys25 and Lys29 residues (observed to interact with Asp192 as shown in **Figure 2**) of the NLS with average distances of 0.3 nm/3 Å (**Supplementary Figure 4C**). Additionally we observed the distances between the conserved Trp184 and the pi interactions observed with Arg33, Arg34, Arg35, and Arg37. Thus, the distance between these residues was calculated by utilizing the center of mass of residues 33–37 in NLS and the CE2 atoms of Trp84, and it was observed to stabilize at 0.5 nm/5 Å (**Supplementary Figure 4D**). This simulation outcome indicates that the NLS interactions with the ARM helices of importin might be stable, thereby further corroborating the prediction that RBPL34 might function as a NM. This indicates that despite the changes observed in importin when analyzed with the complete protein RBPL34, the interacting residues between NLS and importin ARM 2, 3, and 4 are retained.

In order to further corroborate the role of the conserved residue Asp192 for binding of NLS in RBPL34, we mutated the Asp192 residue to an Ala192 residue and docked and minimized the generated complex. Furthermore, this mutated complex was also subjected to an explicit solvent simulation in order to understand the stability of the docked mutated complex. The docking results showed a reduction in overall HADDOCK score from –125.53 in the unmutated complex to –67.22 in the mutated complex. The clusters obtained by this docking as well as the energy landscape has been provided in **Supplementary Table 5** and **Supplementary Figure 5**. Based on the analysis, we chose Cluster 2 for further simulations. The snapshots and distance calculations between the conserved importin residues as well as the RBPL34 NLS were calculated as discussed before in this complex as well. The results showed that the distances between the conserved residues and NLS increased across the simulation, and the final posture of RBPL34 in the mutated complex did not form favorable interactions with the NLS (**Supplementary Figure 6**). The simulation of the mutated complex shows that the important electrostatic interactions as well as pi interactions between ARM repeats and NLS are not observed in the mutated complex, indicating that the predicted NLS and interactions with acidic patch on importin might be necessary for a stable complex. These results can be further corroborated by experimentally mutating the protein and elucidating the change in nuclear import, which is not part of the scope of the present manuscript. The posture of NLS sequence observed across the simulation places Arg41 close to the Ala CB atom, indicating a hydrophobic interaction. Thus, no electrostatic contacts or interactions with conserved Trp residues in ARM helices are observed in the mutated complex (**Supplementary Figure 6**, **Supplementary Table 6**).

Thus, our *in silico* studies indicate that the *Fn* protein RBPL34 possesses all features of an NM and might be capable of entering the host nucleus. In addition to our findings, other groups have also indicated the role of ribosomal proteins as NMs using experimental studies. Lu et al. (2024) observed ribosomal proteins acting as NMs in *Mycoplasma* (Lu et al., 2024) and indicated that these proteins may be secreted into extracellular vesicles, which can further carry and release them into host cells across different organs. The proteins in host cells can then enter the nucleus to exert effects on host gene expression (Lu et al., 2024). Furthermore, RBPL34 has also been observed in the EV proteome of *Fusobacterium*, indicating its transfer to host cells through extracellular vesicles. It has been suggested in recent studies that *Fusobacteria* also form extracellular vesicles that aid in colonization and have also been reported to be associated with CRC progression (Zheng et al., 2024). In addition, studies have also implicated eukaryotic RBPL34 in influencing cancer progression (Luo et al., 2016; Liang et al., 2023). Although these inferences from the *in silico* sequence and structural analysis indicate that RBPL34 in *Fusobacterium* might influence disease progression by entering the host cell nucleus, they can be further corroborated by conducting laboratory experiments to assess the localization of these proteins in the host.

### Homologs of protein TnpB as potential nucleomodulins in *Fna*

The NLS prediction tools identified the presence of cNLS sequence as EKTGKFKKKKRFGKSL consisting of residues 344–359 of the protein with GenBank accession ID WYD17513 in the chosen strain of *Fna*. The protein sequence was conserved (100% identity) across the homologs in *Fna* Clade C2 and was observed to co-occur with the gene encoding TnpA. The analysis of the protein using the Conserved Domain Database (https://www.ncbi.nlm.nih.gov/cdd/) as well as HHpred (https://toolkit.tuebingen.mpg.de/tools/hhpred) indicates the protein (WYD17513) to be a homolog of TnpB, which is a family of proteins observed in IS200/605 and IS607 transposons in bacteria and in many cases, as observed in *Fna*, flanks the TnpA transposase protein (Karvelis et al., 2021). Although the TnpB was earlier considered to be an accessory protein to transposase TnpA, it has now been characterized as an RNA-guided DNA nuclease and is also considered to be an ancestor of CRISPR-associated Cas nucleases (Karvelis et al., 2021). The TnpB protein is considered important in retaining the transposon in a bacteria post-ligation (Karvelis et al., 2021). Studies show that there is a non-coding RNA observed within the same locus as TnpB proteins called omegaRNA or ωRNA, which aids in RNA-guided cleavage of DNA substrates. The 3′ end of the transposon is also called the right end (RE) and demarcates the 3′ end of the ωRNA. The RE is followed by a guide sequence encoded outside of the transposon that enables recognizing the target sequence for cleavage (Karvelis et al., 2021; Nety et al., 2023). The TnpB protein observed in *Fusobacterium* is followed by a non-coding 200-nucleotide inter-genic region indicating the possibility of ωRNA and guide RNA presence in this case as well.

Interestingly, the homologs for this *Fusobacterium* TnpB were observed in plasmids from gut pathogens like *Clostridium botulinum* and *Clostridium difficile* with two to three mismatches in the NLS where FKK residues are substituted with residues INR in the plasmid. Similar changes in NLS are also observed in viral homologs from *Caudovirales* as well as *Clostridium* phages. Despite changes in the sequence, cNLS was also predicted within these proteins by the NLS prediction tools indicating that the homologs also retain the capacity for nuclear localization. On further analysis using HHpred and BLAST, the results showed that the NLS region was observed as an insertion within the *Fusobacterium* and *Clostridium* RNA-guided endonucleases TnpB. The region corresponding to NLS was not observed in experimentally characterized TnpB structures as well as in HMMs used for guided TnpB proteins catalogued in Conserved Domain, TIGRFAM, and PFAM databases (**Supplementary Figure 7**). All these observations may suggest that TnpB proteins with insertion of NLS sequences observed in gut pathogens like *Fusobacterium* and *C. difficile* might lead to their entry into the nucleus and influence gene expression. Our results show insertions of NLS in currently available genome sequences of bacteria *C. botulinum*, *C. difficile*, and *Fusobacterium* and the viral homologs in *Fusobacterium* and *Clostridium* phages. Despite this, it is possible that the TnpB sequence with NLS might also be observed in other bacteria due to horizontal transfer as the protein evolves.

Experimental studies have shown that fusing an NLS sequence with a TnpB with no NLS of its own showed significant changes in the host nucleus (Karvelis et al., 2021). The authors designed plasmids encoding the TnpB protein and fused it with the nuclear localization sequence and reRNA sequences, and these constructs were transfected into human HEK293T cells (Karvelis et al., 2021). The targeted cleavage sites in the experiment showed mutations at frequencies of 10%–20%, thereby indicating repair events and genome editing (Karvelis et al., 2021). Thus, this study showed that the presence of NLS in the protein TnpB provides it the capability of human cell genome editing, indicating its potential use in gene editing as a simpler alternative to CRISPR-Cas. In addition to having a nuclear localization property, an NM needs to be released out of the *Fusobacterium* and reach the host cells to exert its effect. Studies in *Acinetobacter baumannii* have indicated the presence of transposases with NLSs, which provides them a nuclear localization property (Moon et al., 2012a). Studies with fused GFP showed its presence in the nucleus of COS-7 cells (Moon et al., 2012a; Moon et al., 2012b). Furthermore, GFP fused with *A. baumannii* transposase showed presence in the nucleus of A549 cells and induced DNA methylation of E-cadherin gene, thereby reducing its expression using epigenetic modifications (Moon et al., 2012a). The study also showed presence of transposases in extracellular vesicles produced by the bacterium through which they could be delivered to host cells and finally targeted to the nucleus (Moon et al., 2012a). Although these observations in other bacteria might be indicative of the packaging of TnpB in *Fusobacterium* EVs and the capability of entering the host nucleus, further experimental studies would be required to prove the nuclear localization as well as gene editing capabilities of TnpB.

**Figure 4:**
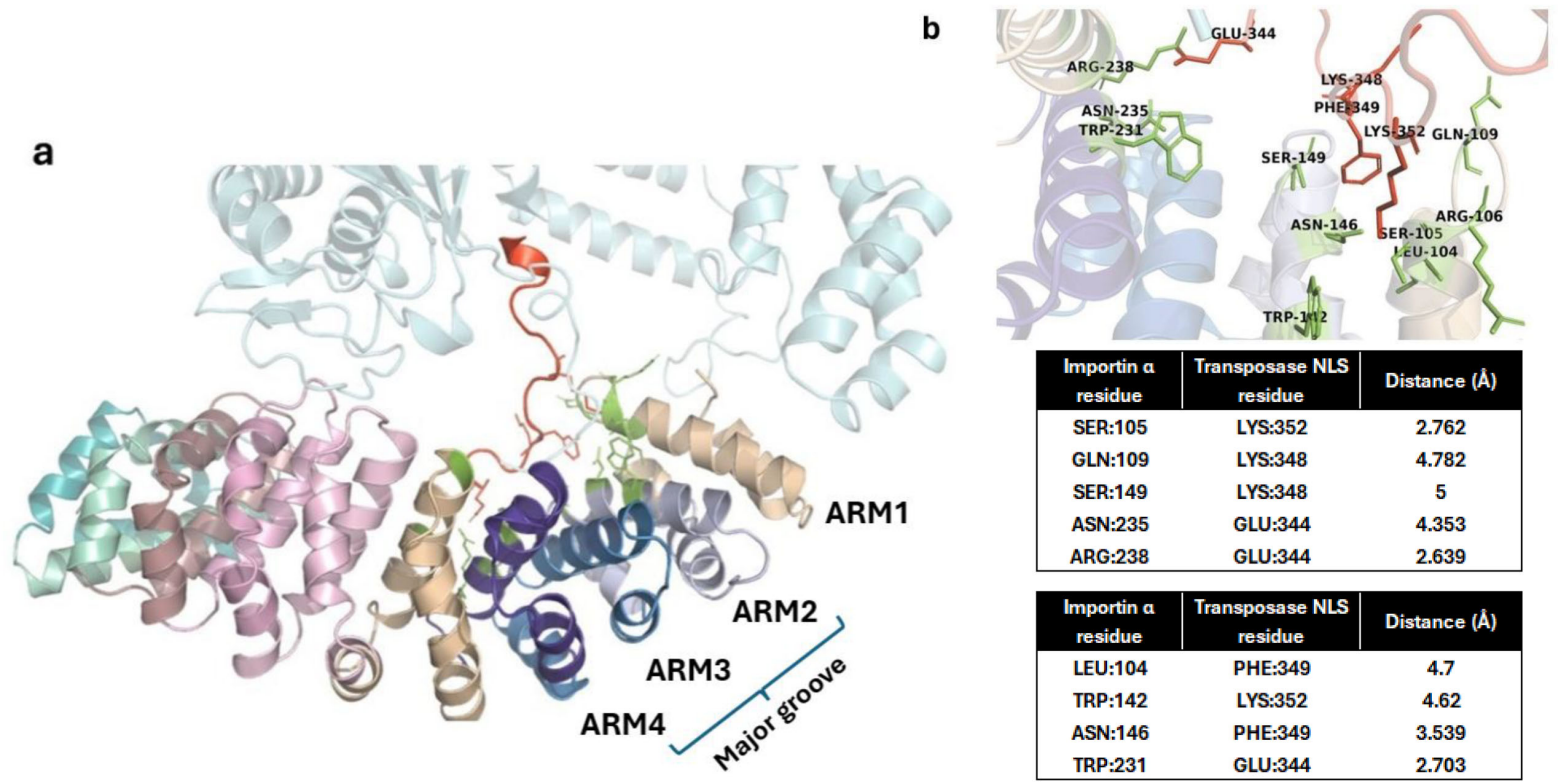
Interaction of importin α with *Fna* SB031 Transposase (Tnp). **(a)** The figure depicts the interaction of NLS in Tnp from *Fusobacterium* with the ARM repeats 1–4 of importin α. **(b)** The cation–pi, hydrophobic, and polar interactions within the docked complex of Tnp and importin α are depicted. The interacting residues from importin are depicted in green while those from Tnp are depicted in red.

In order to further assess the binding of NLS in transposase with importin α, the interactions between the two proteins were assessed using protein–protein docking studies (described in the Methods section). The docked complex showed a HADDOCK score of −100.337 where the TnpB cNLS showed electrostatic, salt bridge, and cation–pi interactions with the importin major groove ARM repeats 1–4 (**Figure 4**). The details of the obtained clusters after the docking of importin-TnpB and their energy landscape have been provided in **Supplementary Figure 8** and **Supplementary Table 7**.

As observed in experimentally characterized importin NLS structures, the Trp142 from ARM 2 and Trp231 from ARM 4 showed cation–pi and hydrophobic interactions with side-chain carbon atoms of Lys352 and Glu344 within the NLS of TnpB protein. Furthermore, the conserved residues Asn146 from ARM 2 and Asn235 from ARM 4 also showed polar interactions with the NLS. In addition to these interactions, the salt bridge is observed between Glu344 in NLS and Arg238 from the importin ARM 4. These interactions are in agreement with those required for cNLS binding to the major groove of importin α. Thus, the insertion of sequence corresponding to predicted NLS in the TnpB from *Fusobacteria* (as compared to Tnp sequences in other bacteria) as well as the interactions observed in the docked complex indicate that this protein might be able to localize in the nucleus and impact gene editing through its endonuclease function specific to certain sequences.

### Other nucleomodulin candidates

A Cas9 CRISPR endonuclease from *Fna* with a cNLS sequence NSRRRLKRRKWRLNLLEEIF was observed in fewer strains of the *Fusobacterium* genus. This indicates that only the homologs of Cas9 with NLS, observed in limited number of strains (of the *Fusobacterium* genus), can be considered as candidate NMs. Homologs possessing the NLS are also observed in bacterial pathogens including *Listeria monocytogenes*, *Treponema*, and *Streptococcus pyogenes* with four to five mismatches in the cNLS sequences. In addition, analysis of gene context of these Cas9 genes in genomes of all these bacteria showed the presence of a complete CRISPR Cas system including the repeat regions.

Another protein observed in a few *Fusobacterium* strains corresponds to WYD16313.1 in *Fna* with predicted cNLS ENLKLIKKKKRYKNSSVYYL corresponding to residues 82–101 on the protein. The homologs of the protein are observed in only a few sequenced strains of *Fna* and *F. polymorphum* with three mutations in the NLS region. Interestingly, the protein has matches with a >50% identity in *Fusobacterium* phages and other human gut phages belonging to the *Caudoviricetes* family. Furthermore, it was observed that the proteins in *Fusobacterium* strains belonging to species other than *animalis* as well as in *Fusobacterium* phages had insertions that were missing in the *Fna* protein. Despite these insertions, the cNLS was retained with just 2 mismatches in all these strains as well. The cNLS prediction tools also indicated the presence of NLSs in these proteins.

Although the protein WYD16313.1 has been annotated as Replication initiator protein A (RepA), the domain-based annotations using HHPred and Interpro (www.ebi.ac.uk/interpro/) indicate the presence of the N-terminal domain (NTD) of RepA in the first 106 residues while no domain predictions are observed for the remaining 170 C-terminal residues. It is known that while the NTD in RepA proteins is in a conserved DNA binding winged helix domain, the C-terminal domain shows significant sequence changes across bacterial genera (Schumacher et al., 2014).

**Figure 5:**
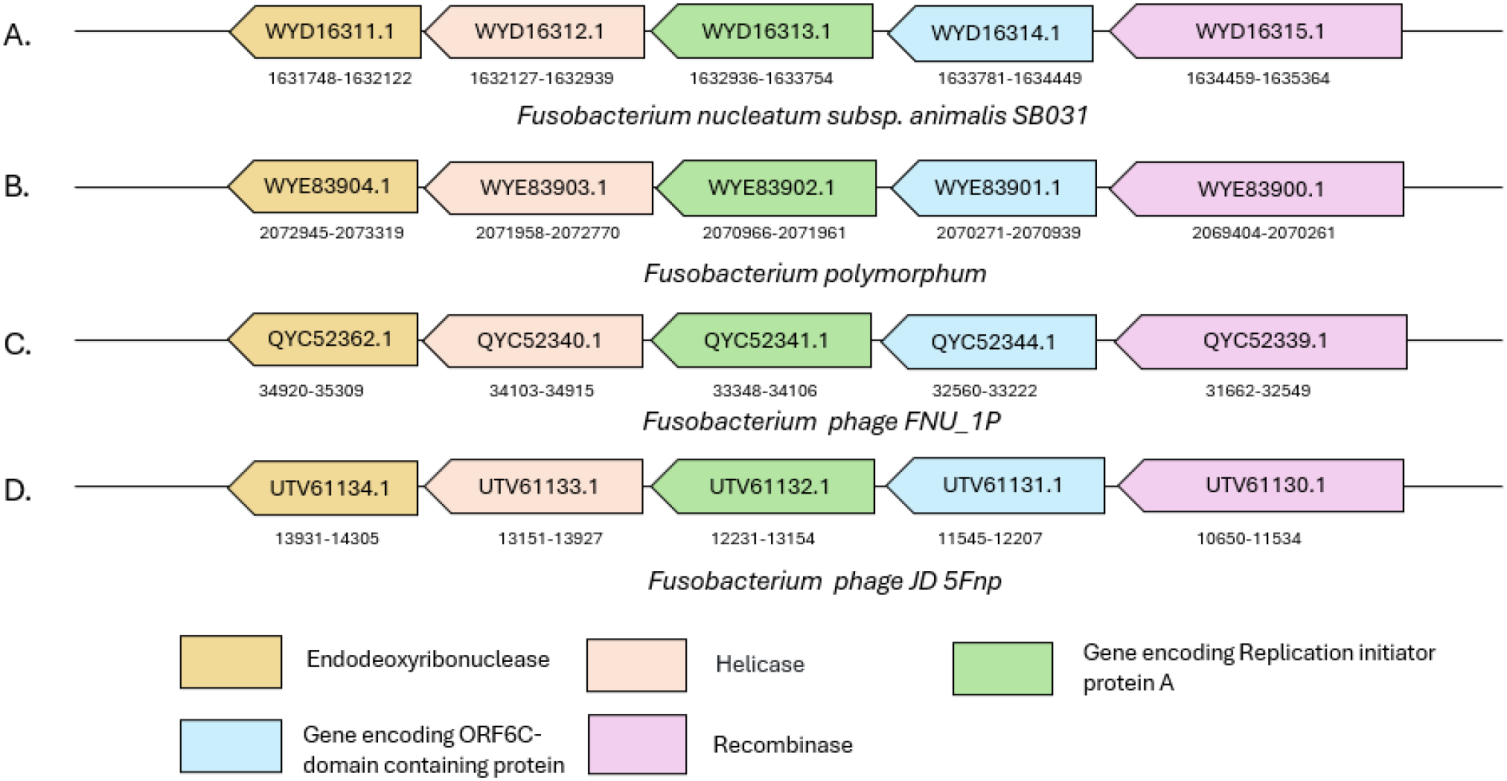
Schematic representation of the genomic context of *repA* in **(A, B)** two *Fusobacterium* strains and **(C, D)** two *Fusobacterium* phages. The genomic locations and GenBank IDs of proteins encoded by the respective genes are mentioned for each of the genomes shown in the figure.

Thus, in order to further corroborate that the protein is indeed a RepA, we analyzed the flanking genes of this protein within the *Fna* strain. Interestingly, the genomic neighborhood of the identified RepA protein in *Fusobacterium* genomes included a helicase and a helicase loader (DnaB and DnaC) protein as well as a large number of recombinase and nuclease proteins. A similar set of genes was also observed in the vicinity of each other in the *Fusobacterium* phages (**Figure 5**). Furthermore, the NLS of Rep proteins in adeno-associated virus (AAV) has been shown to aid in the translocation of these proteins to the nucleus of infected human cells (Cassell and Weitzman, 2004). Thus, the homologs of RepA (in *Fna*) identified in the current study may have the potential to translocate to host cell nucleus with the help of their predicted cNLS, which can further be experimentally validated.

The protein RepA has been characterized in plasmids and is known to initiate replication using its winged helix domain for DNA binding. The protein finally binds the primase helicase DnaB/DnaC for the initiation of plasmid replication (Wegrzyn et al., [[NoYear]]). Thus, the observations indicate that the winged helix domain within the *Fusobacterium* RepA might interact with human DNA after entering the human nucleus. The role of the winged helix domain in human transcription and its effect on DNA-bound complexes have already been observed in earlier studies (Harami et al., 2013). Furthermore, the studies show that the characterized RepA proteins interact with DnaB and DnaC proteins to initiate replication, and the recombinase and nuclease proteins are utilized to bring about variations in the target DNA. Thus, all these proteins are shown to create a complex that can help in the initiation of plasmid replication. Therefore, it is possible that these proteins can form a complex and be carried together as a cargo using the RepA protein’s NLS to enter the human nucleus. The role of these proteins in human cells needs to be explored using further experimental studies.

## Discussion

Various strains of *Fn* have been implicated in the initiation and progression of CRC, oral cancer, and other oral conditions in various stages (Bullman et al., 2017). Even though the studies do not provide affirmation on *Fusobacterium* being a causal organism, it is certainly involved in accelerating the pathophysiology and spread of disease and is also correlated with malignant phenotypes (Bullman et al., 2017; Udayasuryan et al., 2024). Thus, it is important to understand *Fusobacterium*’s interactions within the microbiome as well as its influence on the host genetic makeup in order to delineate its role in exacerbating symptoms.

One of the mechanisms by which bacteria control host machinery is the presence of NMs as explored by this study. The NMs identified in bacteria are required to satisfy two criteria in order to reach the host nucleus. One involves moving out of the bacterial cell into the host environment and then being released into the host cells where it can target the nucleus (Hanford et al., 2021). In our study, we observed potential NMs in *Fusobacterium* possessing the cNLS signature, which indicates its potential to bind the importin α through its major groove. In this study, we have provided *in silico* analysis through molecular docking and simulations to indicate features on identified candidates that could aid in binding the importin. These computational inferences can be further confirmed by an experimental analysis using GFP-tagged proteins (beyond the scope of this study) to assess their nuclear entry on CRC human cell lines. This addition to the candidate NM repertoire might shed more light on the overall impact of the bacterial proteins on host genetic and epigenetic modulation.

Although the study was initiated with a representative strain from *F. nucleatum* subsp. *animalis* Clade C2, we observed homologs of the identified candidate NMs in other subspecies of *F. nucleatum* including *F. nucleatum* subsp. *polymorphum* (*Fnp*) and *F. nucleatum* subsp. *nucleatum* (*Fnn*). It was further observed in scientific literature that the role of *Fnp*, *Fnv*, and *Fnn* has been reported in several oral disease conditions including oral cancer. These studies suggested the involvement of the above subspecies in influencing the expression of host inflammatory genes (Ponath et al., 2022; Wolf et al., 2025). Thus, the presence of NLS containing RBPL34 in these subspecies (as mentioned in Results), in addition to *Fna*, may suggest its involvement as an effector NM in the pathophysiology of oral diseases as well. Furthermore, among the other identified candidate NMs, TnpB and RepA can be expected to have a phage origin as homologs are observed in only *Fusobacterium* and *C. difficile* and *C. botulinum* phages. Studies have shown that these pathogens also influence host gene expression (Miao et al., 2017; Tsakiroglou et al., 2024). Thus, in addition to *Fusobacterium*, if the sequence with the NLS corresponding to these proteins is proven to be a functional NLS and is horizontally transferred to other bacteria, the recipient will also show the nuclear entry phenotype. The aim of this study does not indicate NMs specific to *Fusobacterium* but indicates the category of proteins (*in silico* findings) that can help bacteria influence the host machinery.

As mentioned, prior to nuclear entry, the NMs need to be released into the host cell. Recently, it has been shown that bacterial extracellular vesicles (EVs) allow for transfer of bacterial cargo across host environment and into host cells, thereby facilitating the host organ–microbiome axis often observed to influence human health and immunoregulation (Zheng et al., 2024). In addition, studies showed that EVs from *Fusobacterium*, when administered orally, show retention in the gastrointestinal tract despite *Fusobacterium* being an oral bacterium (Zheng et al., 2024). The *Fn* EVs have been shown to initiate or accelerate proinflammatory responses in the host in multiple organs. These literature reports, along with the insights from the current study, may suggest the importance of EVs in oral-gut communication and probability of involvement of EVs in translocation of the predicted NMs into host cells.

Recent studies have shown that *Fusobacteria* EVs (FEV) are enriched within CRC tissues and increase the colonization of *Fusobacterium* as well as the progression of CRC. The studies show a membrane fusion between human cells and *Fn* EVs that brings about the transfer of cargo to host cells (Zheng et al., 2024). An earlier study identified ribosomal proteins with the capability of nuclear localization in *Mycoplasma (* Lu et al., 2024). Furthermore, a recent proteomic study on *Fusobacterium* EVs obtained from individuals with CRC indicated the presence of RBPL34 in FEVs (Zheng et al., 2024). Similarly, a transposase, experimentally characterized to be an NM in *A. baumannii*, was identified within EVs of this bacterium (Moon et al., 2012a). Identification of similar proteins from *Fusobacterium* as potential NMs in this study can provide a lead to assess their presence in FEV proteome. Although the presence of transposase-like proteins has been observed in earlier studies in FEVs, their role as a TnpB homolog is not ascertained. Thus, an in-depth analysis of *Fusobacterium* EV proteome might give more insights into the action of these proteins within the host. Animal studies on GFP-tagged proteins can be further used to ascertain the complete path of these identified NMs from *Fusobacterium* into the host nucleus.

## Conclusion

The results of the current study including the identification of candidate NMs in *Fn* and predicting their interaction with importin alpha have been performed through extensive sequence (*Fn* genomes) and structural analysis (docking and MD simulation). The study provides a catalog of proteins of *Fn* with strong propensity to act as NMs, which may further be explored for their role in the pathophysiology of diseases like cancer. The insights obtained from the current study would help in designing future experiments involving *in vivo* and *in vitro* models towards understanding the mechanism of translocation of these NMs into the host nucleus and their subsequent mode of action.

## Data Availability

The original contributions presented in the study are included in the article/**Supplementary Material**. Further inquiries can be directed to the corresponding author.
