# Supplementary material for: Can Fusobacterium utilize nucleomodulins in the pathophysiology of CRC?
Source: Front Cell Infect Microbiol. 2025 Oct 30;15:1644443. doi: 10.3389/fcimb.2025.1644443 (PMC12611965; doi:10.3389/fcimb.2025.1644443)
Supplement: Supplementary file 2 [file DataSheet2.pdf]

Supplementary Material

Table of contents

**S-I**	Average Nucleotide Identity (ANI) values between 65 genomes belonging to *F. nucleatum subsp. animalis*
**S-II**	Prediction of secondary structure of known nucleomodulins
**S-III**	Energy landscape and conformational diversity in importin-RBPL34 docked models
**S-IV**	Molecular dynamics simulations of docked complex of importin and RBPL34
**S-V**	Energy landscape and conformational diversity in mutated importin-RBPL34 docked models
**S-VI**	Contacts with conserved Trp residues in ARM helices in mutated importin-RBPL34 complex
**S-VII**	Predicted Nuclear Localization Signal (NLS) missing in other bacterial TnpB homologs
**S-VIII**	Energy landscape and conformational diversity in importin α and Tnp docked models

S-I: Average Nucleotide Identity (ANI) values between 65 genomes belonging to *F. nucleatum* subsp. *animalis*

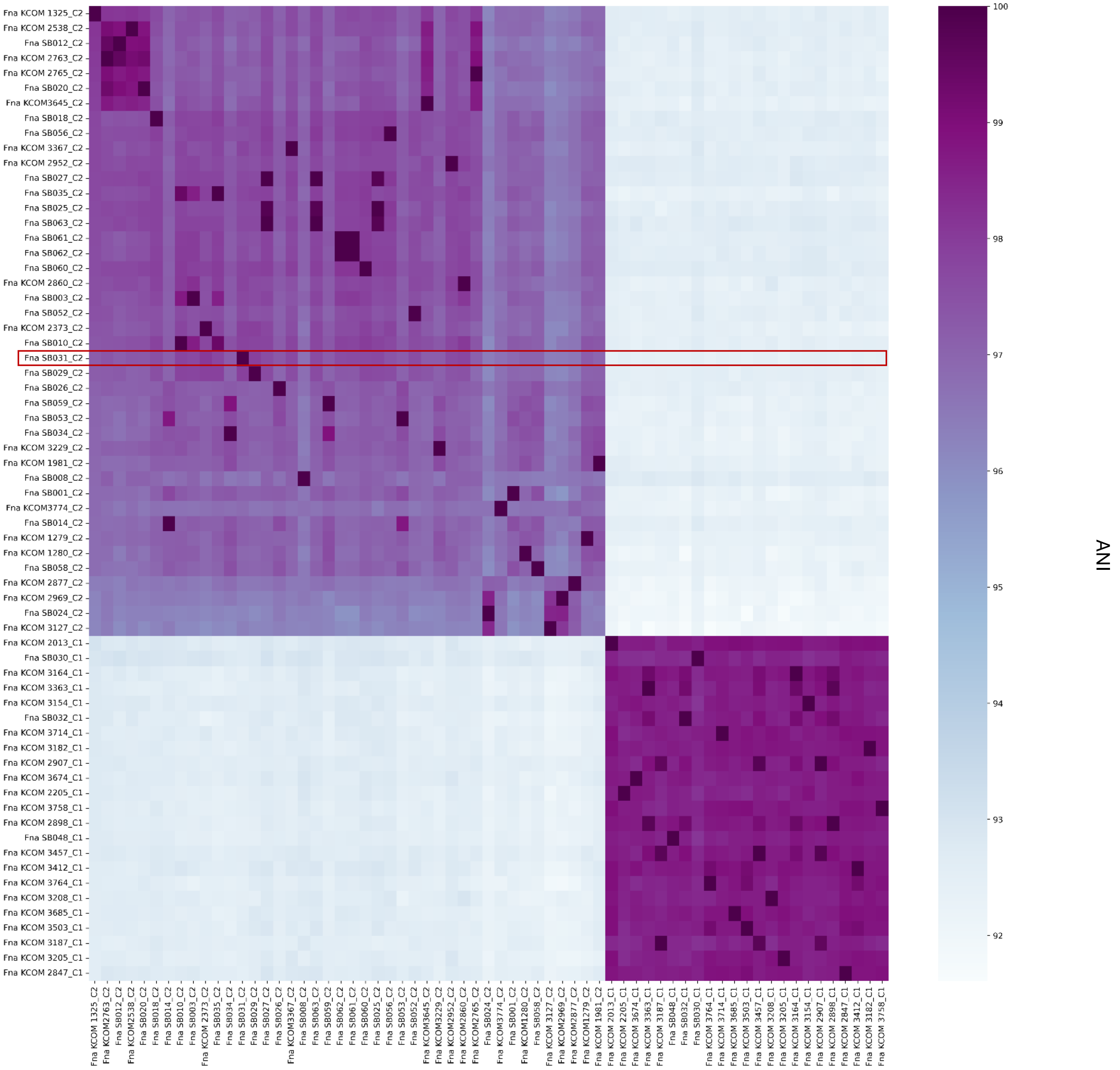

Supplementary Fig 1. Above heatmap represents the ANI values calculated using FastANI (https://github.com/ParBLiSS/FastANI) for pairwise comparisons of 65 nucleotide sequences belonging to *Fna* Clade1 and Clade2. Each cell in the heatmap reflects the pairwise ANI values generated between the sequences. The color gradient indicates the degree of genetic similarity between the sequences, with darker shades corresponding to higher ANI values.

S-II. Secondary structure prediction of known nucleomodulins

i. **Outer membrane protein 18 (Omp18)** from *Helicobacter pylori* 266695 (Accession Id: WP_001037790.1)

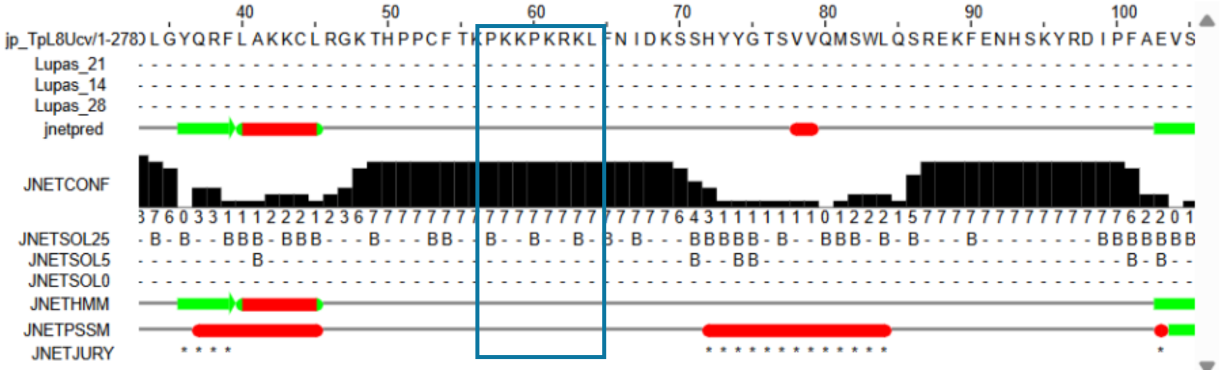

ii. **Terminus utilization substance (Tus)** from *Escherichia coli* (Accession Id: AAA82083.1)

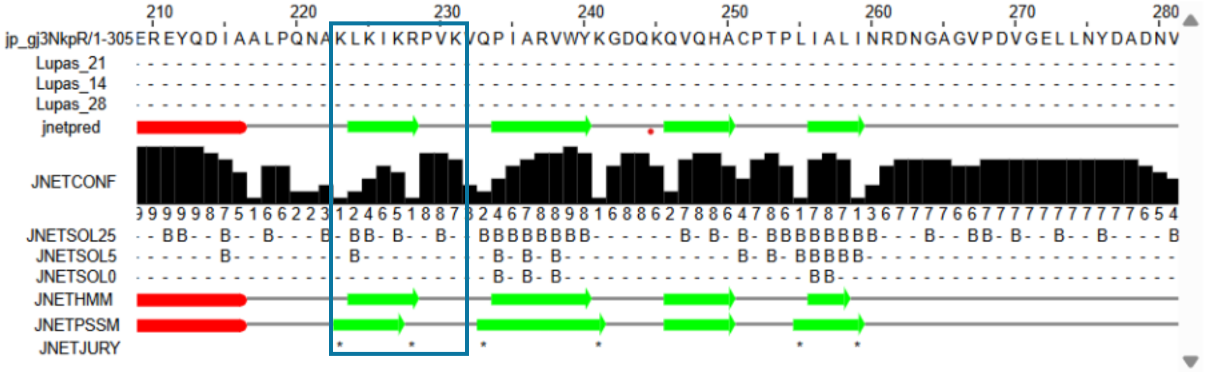

iii. **HP0425** from *Helicobacter pylori* (Accession Id: AAD07494.1)

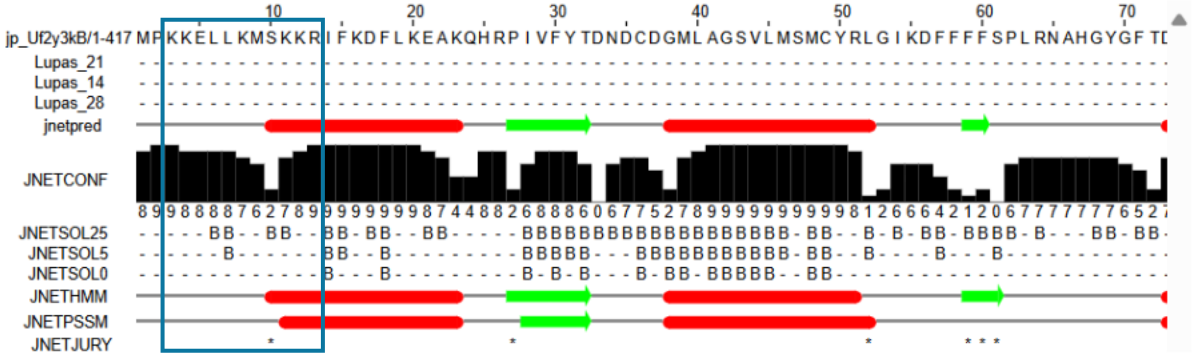

iv. **Cas9** from *Campylobacter jejuni* (Accession Id: WP_058001872.1)

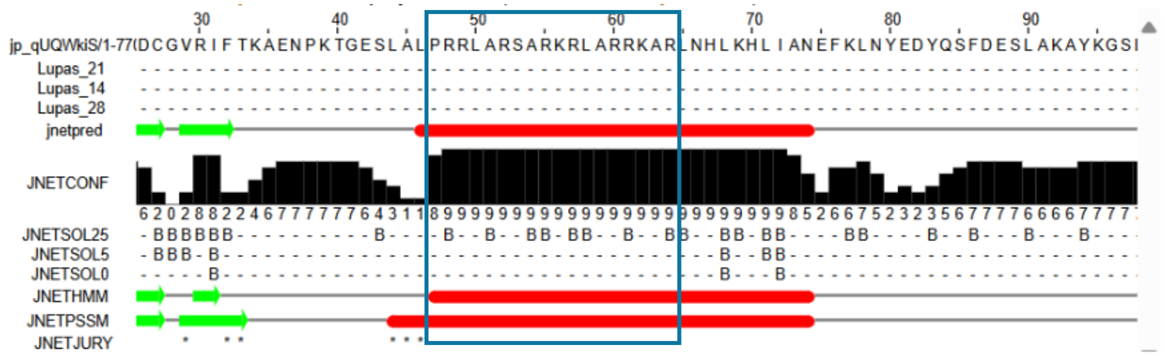

v. **HsdM** from *Klebsiella pnemoniae* (Accession Id: AAB70709.1)

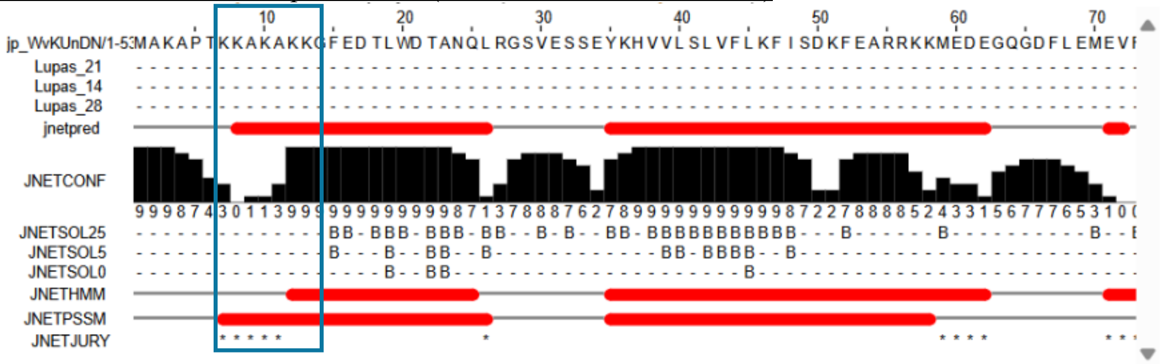

vi. **UreA** from *Helicobacter pylori* 26659 (Accession Id: WP_000779223.1)

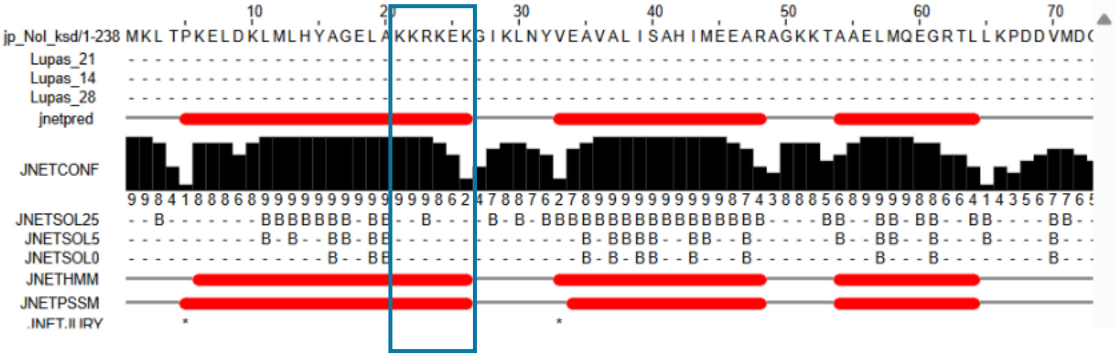

vii. **Transposase Tnp** from *Acinetobacter baumanii* (Accession Id: WP_011858087.1)

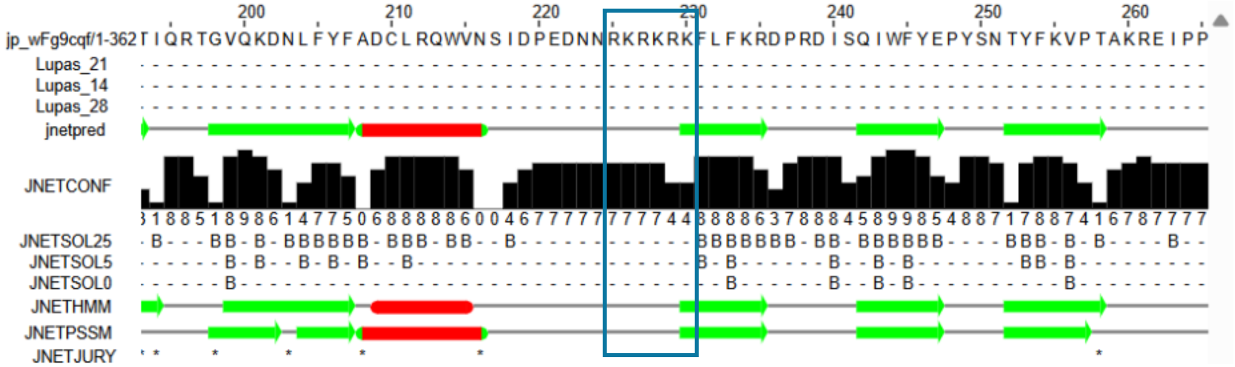

Supplementary Fig 2. Above are images of secondary structures predicted by JPred 4. The output, JNetPRED depicts consensus prediction for α-helices, β-strands in red tubes and green arrows respectively. The region

highlighted in blue box indicates the NLS which has been experimentally verified in various studies. The confidence level for the prediction is provided by JNETCONF with high values corresponding to higher confidence.

S-III. Energy landscape and conformational diversity in importin-RBPL34 docked models

(a)

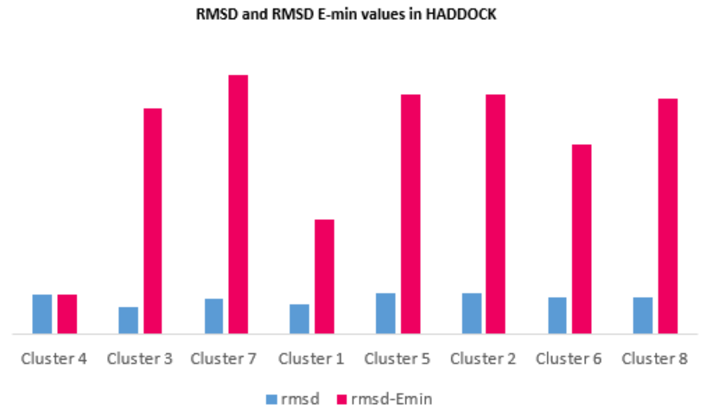

(b)

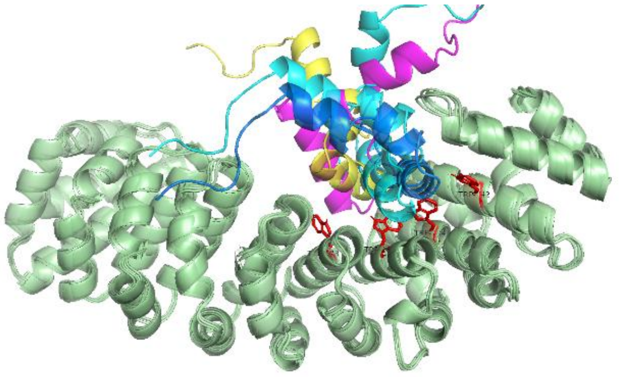

Supplementary Fig 3. (A) Bar plot of average Root Mean Square Deviation (RMSD) and RMSD-Emin for the top clusters of importin-RBPL34 docked models **(B)** Superimposed structures of the representative models from 4 top-ranked clusters with importin represented in palegreen color. RBL34 in each complex is shown in a different color.

S-IV. Molecular dynamics simulations of docked complex of importin α and RBPL34

A. Radius of gyration (Rg)

Replicate1

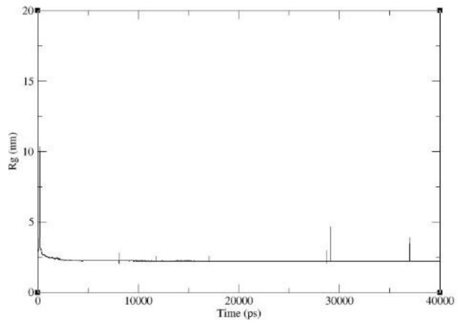

Replicate 2

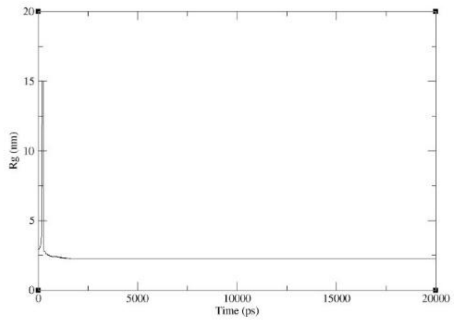

B. Secondary structure frequencies across simulation

Replicate 1

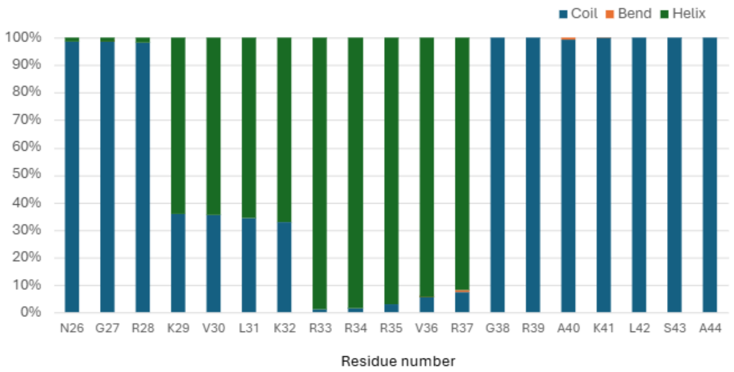

Replicate 2

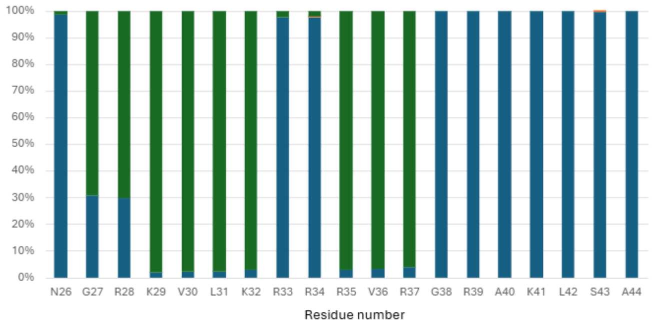

C. Distance across the simulation between residues OD2 of Asp 192 of importin and NZ of Lys 29 of NLS

Replicate 1 Replicate 2

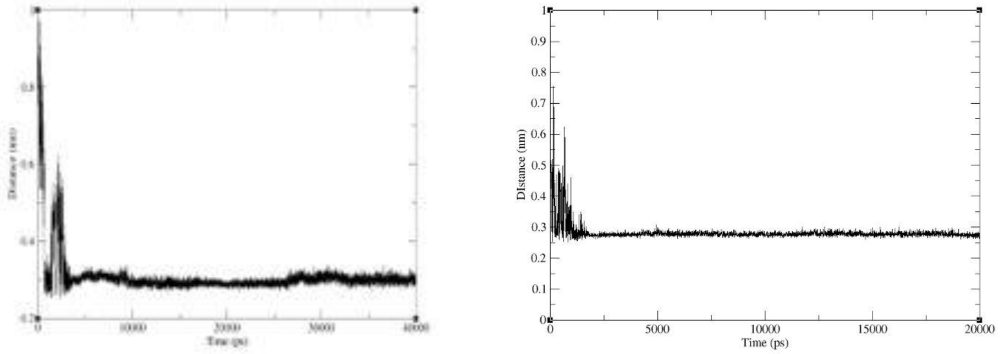

D. Distance across the simulation between residues Trp 184 of importin and Center of Mass of Res 33-37 of NLS

Replicate 1 Replicate 2

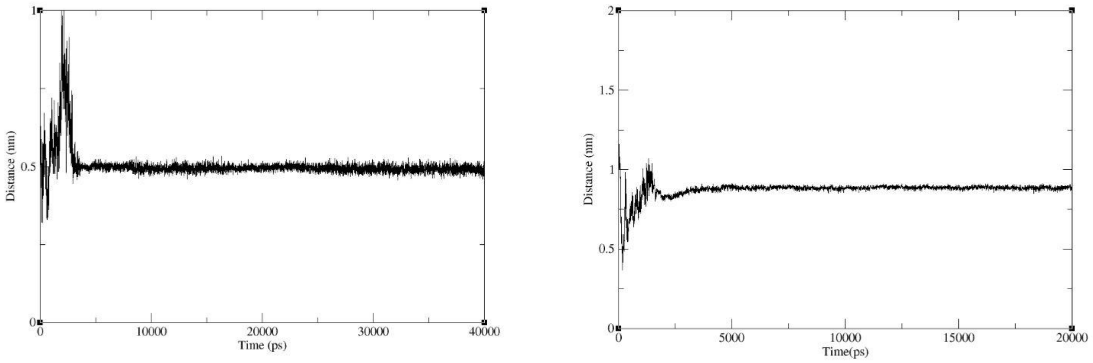

Supplementary Fig 4. (A) Radius of gyration (for both simulation replicates) showing the reduction in first 2ns followed by stabilization of Rg value. **(B)** Conformational changes in secondary structure of NLS region across simulation **(C)** and **(D)** Distance across the simulation between residues of importin and NLS.

S-V. Energy landscape and conformational diversity in mutated importin-RBPL34 docked models

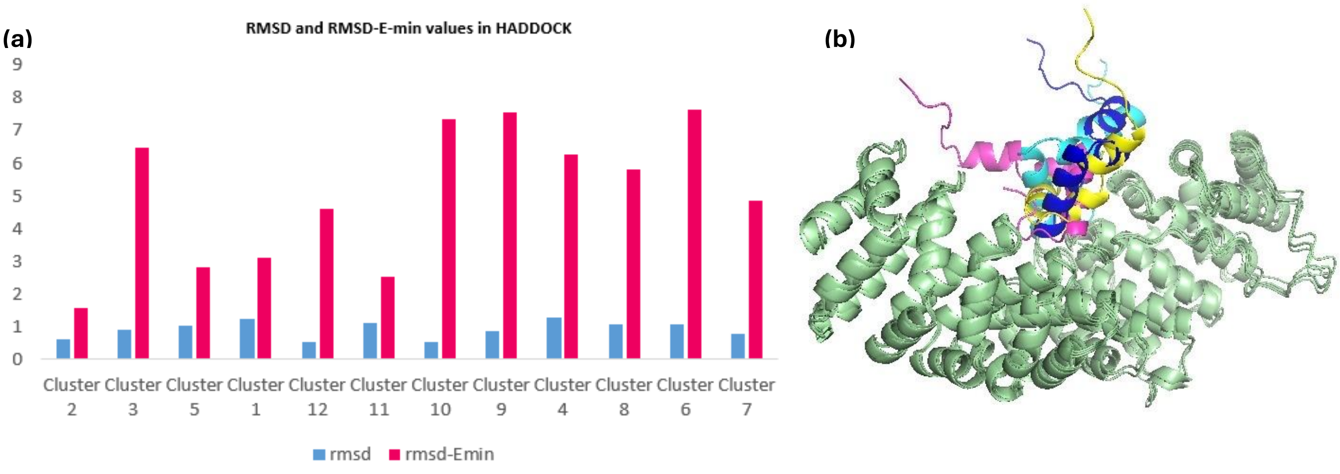

Supplementary Fig 5. (A) Bar plot of average Root Mean Square Deviation (RMSD) and RMSD-Emin for the top clusters of mutated importin (Asp192Ala)-RBPL34 docked models **(B)** Superimposed structures of the representative models from 4 top-ranked clusters with importin represented in palegreen color. RBL34 in each complex is shown in a different color.

S-VI. No contacts with conserved Trp residues in ARM helices in mutated importin-RBPL34 complex

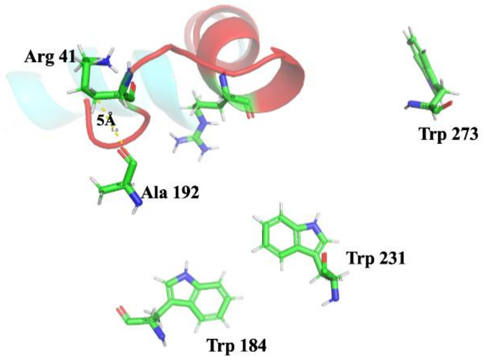

Supplementary Fig 6. Loss of contacts in the mutated importin-RBPL34 complex upon 20ns simulation

S-VII. Predicted Nuclear Localization Signal (NLS) missing in other bacterial TnpB homologs

1. **8H1J_A RNA-guided DNA endonuclease TnpB; CRISPR-Cas, Transposon-associated protein, RNA BINDING PROTEIN;{Deinococcus radiodurans R1 = ATCC 13939 = DSM 20539}**

Probability: 100%, E-value: 1.8e-39, Score: 325.64, Aligned cols: 359, Identities: 19%, Similarity: 0.2, Template Neff: 11.3

```
Q ss_pred              CCeEEEEEEEeeCCHHHHHHHHHHHHHHHHHHHHHHHHHHHHHHHHHhcCHHHHHHhhccHHHHHHHHHHHHHHhCCCHHHH
Q WXZ65270.1    1     MANYVLTLALKTELWQEHILEKRLNIARMIYNSCLSEILKRHRKMINSSEYKGISNLDKKEQSKRYKELDKKYLISKFEL   80 (475)
Q Consensus     1     ~~~~t~k~rl~p~~~q~~~L~~~~~~~~r~~yN~~l~~~~~~~~~~~~~~~~~~~~~~~~~~~~~~~~~~r~~~~~~~~~l   80 (475)
                      |+++|++++|+|+++|...|..++..++++||.++++..+.+.....                       .+++.++
T Consensus     4     ~~~rt~~~~l~~~~~q~~~l~~~~~~~~r~~~N~~l~~~~~~~~~~~~-------------------------~~~~~~~   57 (410)
T 8H1J_A        4     IRNKAFVVRLYPNAAQTELINRTLGSARFVYNHFLARRIAAYKESGK-------------------------GLTYGQT   57 (410)
T ss_dssp             EEEEEEEEEEECCHHHHHHHHHHHHHHHHHHHHHHHHHHHHHHHSC-------------------------CCCHHHH
T ss_pred              eeeEEEEEEEeCCCHHHHHHHHHHHHHHHHHHHHHHHHHHHHHHhCC-------------------------CCChhhc

Q ss_pred              HHHHHHHHH----HHHhccChHHHHHHHHHHHHHHHHHHHh------CCCCccEEeccccceeEEecCCCCceeEEEecCCCE
Q WXZ65270.1    81    NKYVKPMTQ----KFKKNIGSQMGQELAERAFATYEKFKY------GKAKKVYFKSYENFYSVREKGNITGLRFFKEDCC   150 (475)
Q Consensus     81    ~~~~~~~~~~~----~~~~~~l~s~~~q~~~~~~~~~~a~k~~~~~------~~~k~P~fk~~~~~~~s~~~~~~~~~~~~~~~~~~   150 (475)
                      ...+...+.    +|+.++|++++|+++.++..+|++|++      ...+.|.|+++....++.+...+.++.+.  ++.
T Consensus     58    ~~~~~~~~~~~~~~~~~~~~l~s~~~q~~~~~~~~~~a~ks~~~~~k~~~~~~~~~~P~~k~~~~~~~~~~~~~~~~~~~~~~~--~~~~   135 (410)
T 8H1J_A        58    SSELTLLKQAEETSWLSEVDKFALQNSLKNLETAYKNFFRTVKQSGKKVGFPRFRKKRTGESYRTQFTNNNIQIG--EGR   135 (410)
T ss_dssp             HHHHHHHHHSSTTGGGGTSCHHHHHHHHHHHHHHHHHHHHHHHHHSCSSCCCCCCCCCCSCEEEEEEECCTTCSEEC--SSE
T ss_pred              hHHHHHHHhhhcCchHhhccHHHHHHHHHHHHHHHHHHHHHHHHHhcCCCCCCCCeeCCCCCCCeEEEecCCCcEEEc--CCE

Q ss_pred              EEECCeeEeeeecCCCHHHHHHhcCCeeEEEEEEEEEECCceeEEEEEEEeCCCCCCccCCCCCCeEEEEeCCCeEEEEEeCC
Q WXZ65270.1    151   ISWLGLKIPVIIKNNDKYAQSCFLDKLLYCRLLKRVVNGKNKYYVQITFEGTPPKKHKVGGENEIGIDIGTSTIAIVSDN   230 (475)
Q Consensus     151   i~l~~~~~~~~~~~~~~~~~~~~~~~~~~~~~~~~~~~~i~~~~~~~g~~~~~y~~i~~~~~~~~~~~~~~~~~~~~~~gID~G~~~~~~~~~s~g   230 (475)
                      +.|+..+. +.....     ..+.+.+.++.|++..    .+.||++++++.+.++. +..+..++|||+|+.+++++++|
T Consensus     136   ~~l~~~~~~-~~~~~~~-----~~~~~~~~~~~~~~i~~~~---~~~~~~~l~~~~~~~~~~~~~~~~~-~~~~~~~~~gID~G~~~~~~~~~~~   205 (410)
T 8H1J_A        136   LKLPKLGW-VKTKGQ-----QDIQGKILNVTVRRIH---EGHYEASVLCEVEIPYL-PAAPKFAAGVDVGIKDFAIVTDG   205 (410)
T ss_dssp             EEETTTEE-EEEESC-----CCCCSEEEEEEEEEEE---TTEEEEEEEEEECCCC-CCCSEEEEEECCCSSEEEEEECS
T ss_pred              EEcCCCCe-eEEEec-----cCCCCCeEEEEEEEEe---CCEEEEEEEEEEecCCC-CCCCCCeeEEeeeCCcEEEEeCC

Q ss_pred              ceeEE-ecccccchhhHHHHHHHHHHHHHHHHHhCccccCCCCCccccCccccccCHHHHHHHHHHHHHHHHHHHHHHHHH
Q WXZ65270.1    231   KVELK-ILAENIEINEKEKTRLQRKLDRQRRANNPNKYNADGTINIENKEKWKKSKSYVKTKLKLSNLQRKIAEKREQSH   309 (475)
Q Consensus     231   ~~~~~~-~~~~~~~~~~~~~~~~~~~~~~~q~~~~~r~~~~~~~~~~~~~~~~~~~~~~~~~~~~~~~~~~~~~~~~s~~~~~~~~~~~~~~~~~~~~~~~~~k~~~~~~~~~~   309 (475)
                      ..+.. ............+..+++.+.+...                       +++++.+....+.++..+..+...+.+
T Consensus     206   ~~~~~~~~~~~~~~~~~~~~~~~~~~~~~~~~~~~~~~~~~~~~~~~~~~~~~---------------------~s~~~~~~~~~~~~~~~~~~~~~~~k~~~~~~~~~~   263 (410)
T 8H1J_A        206   VRFKHEQNPKYYRSTLKRLRKAQQTLSRRKK---------------------GSARYGKAKTKLARIHKRIVNKRQDFL   263 (410)
T ss_dssp             SCEEEEECCTTHHHHHHHHHHHHHHHHHHSCT---------------------TCHHHHHHHHHHHHHHHHHHHHHHHHHH
T ss_pred              cccCCcCChHHHHHHHHHHHHHHHHHHHhhcC---------------------CCccHHHHHHHHHHHHHHHHHHHHHHHH

Q ss_pred              HHHHHHHHHhCCeEEEEeCCCHHHHHhhhcccccccccCccccccccChhHHhhCHHHHHHHHHHHHHHcCCCcEEEEcccc
Q WXZ65270.1    310   NILANSILEIGTIVKVENMSFKGLQRRSKKTEMSEKTGKFKKKKRFGKSLSNRAPALLIEIINRKLEYIGKNIIKIDTFK   389 (475)
Q Consensus     310   ~k~~~~~l~~~~~~~~i~ve~l~~~~~~~~~~~~~~~~~~~~~~~~~~~~~~~~~~~~~~~~~~~~r~l~~~~~~~~~~~~~~~~~~l~~k~~~~~G~~~~~~v~~~~~   389 (475)
                      |++++.|+..+..|++|++....+....                     ..++.+.|.|+++.|...|+++|.++|+.++.|+  +
T Consensus     264   ~~~~~~~~l~~~~~~~~i~ve~l~~~~~~~~~~~~~~~~~~~---------------------~~~~~~~~~~~~~~~~~~~~~~l~~k~~~~~G~~v~~v~--~   325 (410)
T 8H1J_A        264   HKLTTSLVREYEIIGTEHLKPDNMRKNR---------------RLALSISDAGWGEFIRQLEYKAAWYGRLVSKVS--P   325 (410)
T ss_dssp             HHHHHHHHHHEEEEEEECC------------------------------CCCHHHHHHHHHHHHHHHHTCEEEEEC--T
T ss_pred              HHHHHHHHhCCeEEECCCChhHhhhcc---------------chHHHHHHhcHHHHHHHHHHHHHHhCCCceEEec--C

Q ss_pred              cc-hhcccCCCCcEEec--CHHHCeEEec--CcEehhHHHHHHHHHhcccCcc
Q WXZ65270.1    390   VK-ASQLNHSTNEYEKK--SLSKRWVEIL--GNKIQRDLYSAFLIKNVKENLE   437 (475)
Q Consensus     390   ~~~-tS~~c~~Cg~~~~~~~~~-~~~~~~~~~~~~~~~C~--g~~~~~~rD~naa~Ni~~~~~~~~~~~~~   437 (475)
                      ++ ||++||.||.....  ....+.|.|+  |...++|+|||.||+++++...
T Consensus     326   ~~~~tS~~c~~cg~~~~~~~~~~~~~~~~~~~~~~~~~~~c~~cg~~~~~~~~~D~naa~ni~~~~~~~~~~~~   378 (410)
T 8H1J_A        326   YFPSSQLCHDCGFKNPEVKNLAVRTWTCPNCGETHDRDENAALNIRREALVAA   378 (410)
T ss_dssp             TCCTTTBCTTTCCBCGGGGSTTCSEEEETTTTEEEEHHHHHHHHHHHHHHHH
T ss_pred              CCCCCCccccCCCCCCCccccCCCcCEEeCCCCCCCcccchhHHHHHHHHHHHHHHHh
```

Name	Accession	Description	Interval	E-value
guided_TnpB super family	cl45887	RNA-guided endonuclease TnpB family protein; This family includes RNA-guided endonuclease TnpB ...	16-431	1.56e-22

RNA-guided endonuclease TnpB family protein; This family includes RNA-guided endonuclease TnpB from IS200/IS605 family elements (NF038281) and IS607 family elements (NF038280), but also many additional proteins. It exhibits homolog to or actually includes some CRISPR-associated (Cas) proteins such as the type V CRISPR-associated protein C2c8. For a long time, TnpB proteins were described as accessory proteins in IS (insertion sequence) elements, present as one of just one or two proteins encoded in the element but not necessary for transposition. The programmable RNA-guided endonuclease TnpB proteins may provide a CRISPR-like, widespread form of phage defense by RNA-guided DNA degradation.

Pssm-ID: 468544 [Multi-domain] **Cd Length:** 384 **Bit Score:** 99.15 **E-value:** 1.56e-22

```
                          10        20        30        40        50        60        70        80
                  ....*....|....*....|....*....|....*....|....*....|....*....|....*....|....*....|
gi 2704939552  16 QEHILEKRLNIARMIYNSCLSEIlKRHRKMINSSEYKGISNLDKKEQSKRYKELDKKYLISKFELNKYVKPMTQKFKKNi 95
Cdd:NF040570   11 QKRELAELFGAARFLYNAALAER-KEAYEKNGKFLSYKALLKKLLTELKKEKELEWLKELSSQALQQALKRLAKAFKNF- 88

                          90       100       110       120       130       140       150       160
                  ....*....|....*....|....*....|....*....|....*....|....*....|....*....|....*....|
gi 2704939552  96 gsqmgqeLAERAFATYEKFKYGKAKKVYFKSYENFYSVREKGNITGLrffkedccISWLGLKIPVIIKNNDKYAQSCFLD 175
Cdd:NF040570   89 -------FKKLKKAGFPRFKSKKKKVPSYTPQSVNKRLRKKRNRKKK--------NGRLKLPKLGGVKLRLSRILPILLD 153

                         170       180       190       200       210       220       230       240
                  ....*....|....*....|....*....|....*....|....*....|....*....|....*....|....*....|
gi 2704939552 176 KLLY-CRLLKRVVNGKNKYYVQITFEGTPPKKHKV-GGENEIGIDIGTSTIAIVSDNKVELKILAENIEInEKEKTRLQR 253
Cdd:NF040570  154 GKGGkIKSVTISKPKKGKYYVSISVEVEVPEPPPKeVTGKVVGIDLGLKNFATLSDGGEKIENPRFLRKK-EKRLRRLQR 232

                         250       260       270       280       290       300       310       320
                  ....*....|....*....|....*....|....*....|....*....|....*....|....*....|....*....|
gi 2704939552 254 KLDRQRRAnnpnkynadgtinienkeKWKKSKSYVKTKLKLSNLQRKIAEKREQSHNILANSILEIGTIVKV--ENMSFK 331
Cdd:NF040570  233 KLSRKLQR------------------KGKGSSNRKKARKKVARLHRKIANQRKDFLHKLSKRLVKEADANNVvvEDLEVK 294

                         330       340       350       360       370       380       390       400
                  ....*....|....*....|....*....|....*....|....*....|....*....|....*....|....*....|
gi 2704939552 332 GLQRRSKKTEMSektgkfkkkkrfgKSLSNRAPALLIEIINRKLEYIGKNIIKIDTFKVKASQLNHSTNEYEKKSLSKR- 410
Cdd:NF040570  295 GMVKNKKKKKLA-------------KSIHDWAFGQLRRMLEYKAEWYGIKVVKVDPAYTSSQCCSCGGHRKEKLLLSCRe 361

                         410       420
                  ....*....|....*....|..
gi 2704939552 411 WV-EILGNKIQRDLYSAFLIKN 431
Cdd:NF040570  362 WTcPECGYTVHRDINAAINILR 383
```

Supplementary Fig 7. The figure shows the NLS region **EKTGKFKKKKRFGKSL** is an insertion (highlighted in red box) with respect to sequences of TnpB observed in other bacteria as observed in HHPred and CDD.

S-VIII. Energy landscape and conformational diversity in importin α and Tnp docked models

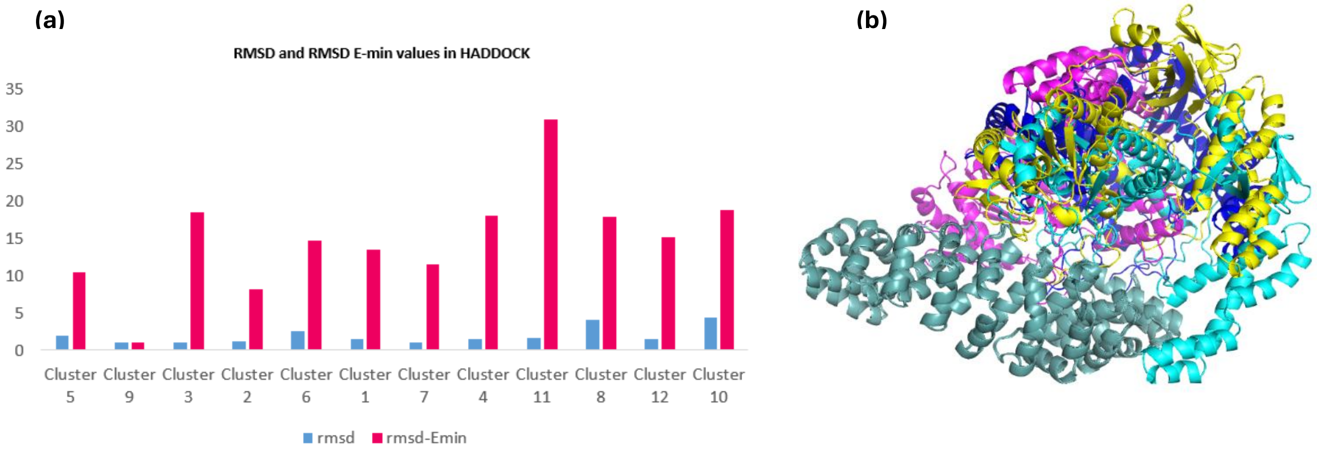

Supplementary Fig 8. (A) Bar plot of average Root Mean Square Deviation (RMSD) and RMSD-Emin for the top clusters of importin-Tnp docked models **(B)** Superimposed structures of the representative models from 4 top-ranked clusters. Tnp in each complex is shown in a different color.

Supplementary References

1. Moon, D. C. *et al.* Nuclear translocation of Acinetobacter baumannii transposase induces DNA methylation of CpG regions in the promoters of E-cadherin gene. *PloS One* **7**, e38974 (2012).

2. Saha, C. *et al.* Guide-free Cas9 from pathogenic Campylobacter jejuni bacteria causes severe damage to DNA. *Sci. Adv.* **6**, eaaz4849 (2020).

3. Saha, C. *et al.* Campylobacter jejuni Cas9 Modulates the Transcriptome in Caco-2 Intestinal Epithelial Cells. *Genes* **11**, 1193 (2020).

4. Kaczmarczyk, S. J., Sitaraman, K., Hill, T., Hartley, J. L. & Chatterjee, D. K. Tus, an E. coli Protein, Contains Mammalian Nuclear Targeting and Exporting Signals. *PLoS ONE* **5**, e8889 (2010).

5. Kwon, Y. C. *et al.* Novel nuclear targeting coiled-coil protein of Helicobacter pylori showing Ca(2+)-independent, Mg(2+)-dependent DNase I activity. *J. Microbiol. Seoul Korea* **54**, 387–395 (2016).

6. Morphological changes in human gastric epithelial cells induced by nuclear targeting of Helicobacter pylori urease subunit A | SpringerLink. https://link.springer.com/article/10.1007/s12275-015-5085-5.

7. Helicobacter pylori HP0425 Targets the Nucleus with DNase I-Like Activity - Kim - 2016 - Helicobacter - Wiley Online Library. https://onlinelibrary.wiley.com/doi/10.1111/hel.12271.

8. Helicobacter pylori Outer Membrane Protein 18 (Hp1125) Is Involved in Persistent Colonization by Evading Interferon- γ Signaling - PubMed. https://pubmed.ncbi.nlm.nih.gov/25945338/.

9. Lee, J. C. *et al.* Prediction of bacterial proteins carrying a nuclear localization signal and nuclear targeting of HsdM from Klebsiella pneumoniae. *J. Microbiol. Seoul Korea* **47**, 641–645 (2009).

10. Pal, R., Ghosh, S. & Mukhopadhyay, S. Moonlighting by PPE2 Protein: Focus on Mycobacterial Virulence. *J. Immunol. Baltim. Md 1950* **207**, 2393–2397 (2021).

11. The PPE2 protein of Mycobacterium tuberculosis translocates to host nucleus and inhibits nitric oxide production | Scientific Reports. https://www.nature.com/articles/srep39706.

12. Besbes, A. *et al.* Hyperinvasive Meningococci Induce Intra-nuclear Cleavage of the NF-κB Protein p65/RelA by Meningococcal IgA Protease. *PLOS Pathog.* **11**, e1005078 (2015).

13. Khairalla, A. S. *et al.* Nuclear trafficking, histone cleavage and induction of apoptosis by the meningococcal App and MspA autotransporters. *Cell. Microbiol.* **17**, 1008–1020 (2015).

14. Hadi, H. A., Wooldridge, K. G., Robinson, K. & Ala'Aldeen, D. A. A. Identification and characterization of App: an immunogenic autotransporter protein of Neisseria meningitidis. *Mol. Microbiol.* **41**, 611–623 (2001).

15. Pennini, M. E., Perrinet, S., Dautry-Varsat, A. & Subtil, A. Histone Methylation by NUE, a Novel Nuclear Effector of the Intracellular Pathogen Chlamydia trachomatis. *PLOS Pathog.* **6**, e1000995 (2010).
